# Excessive proliferation and impaired function of primitive hematopoietic cells in bone marrow due to senescence post chemotherapy in a T cell acute lymphoblastic leukemia model

**DOI:** 10.1186/s12967-015-0543-8

**Published:** 2015-07-17

**Authors:** Chuanhe Jiang, Xiaoxia Hu, Libing Wang, Hui Cheng, Yan Lin, Yakun Pang, Weiping Yuan, Tao Cheng, Jianmin Wang

**Affiliations:** Institute of Hematology, Changhai Hospital, Second Military Medical University, 168 Changhai Road, Shanghai, 200433 China; State Key Laboratory of Experimental Hematology, Institute of Hematology and Blood Disease Hospital, Chinese Academy of Medical Sciences and Peking Union Medical College, 288 Nanjing Road, Tianjin, 300020 China; Center for Stem Cell Medicine, Chinese Academy of Medical Sciences and Peking Union Medical College, Tianjin, 300020 China

**Keywords:** Primitive hematopoietic cells, Leukemia, Chemotherapy, Senescence

## Abstract

**Background:**

In clinic settings, rel apsed leukemic patients are found to be more fragile to chemotherapy due to delayed or incomplete hematopoietic recovery, and hematopoiesis of these patients seem to be impaired.

**Methods:**

We established a leukemia therapy model with a non-irradiated T cell acute lymphoblastic leukemia mouse model combined with cytarabine and cyclophosphamide. Dynamic kinetics and functional status of both primitive hematopoietic cells and leukemic cells in a leukemia host under the chemotherapy stress were comprehensively investigated.

**Results:**

We successfully established the leukemia therapy model with T lymphoblastic phenotype. After treatment with cytarabine and cyclophosphamide, the frequency of L^−^K^+^S^+^ hematopoietic cells tides with the therapy, and stabled when the disease remission, then reduced when relapsed, while leukemic cells showed a delayed but consistent regeneration. Combination of chemotherapy significantly promote an early and transient entrance of L^−^K^+^S^+^ hematopoietic cells into active proliferation and induction of apoptosis on L^−^K^+^S^+^ cells in vivo. Moreover, in the competitive bone marrow transplantation assays, hematopoietic cells showed gradually diminished regenerative capacity. Testing of senescence-associated beta-galactosidase (SA-β gal) status showed higher levels in L^−^K^+^S^+^ hematopoietic cells post therapy when compared with the control. Gene expression analysis of hematopoietic primitive cells revealed up-regulated *p*16, *p*21, and down-regulated *egr1* and *fos*.

**Conclusion:**

We conclude that primitive hematopoietic cells in bone marrow enter proliferation earlier than leukemic cells after chemotherapy, and gradually lost their regenerative capacity partly by senescence due to accelerated cycling.

**Electronic supplementary material:**

The online version of this article (doi:10.1186/s12967-015-0543-8) contains supplementary material, which is available to authorized users.

## Background

Leukemia is the most common hematopoietic malignancy, characterized by uncontrollable growth of leukemic cells and gradual exhaustion of normal hematopoiesis. It is well known that hematopoiesis is a hierarchical system, with primitive hematopoietic cells as ancestors, usually named as hematopoietic stem/progenitor cells (HSPCs). Previously we have revealed kinetics of these cells in an irradiated acute T cell lymphoblastic leukemia (T-ALL) mice model [1]. We found that primitive hematopoietic cells preserved their regenerative capacity in a quiescent cell cycle state even when the numbers decreased. The fate of these cells was more likely depended on the changes of micro-environment rather than cells themselves. In fact, in early 2008, researchers found that leukemic cells could create a special environment that affected maintenance of primitive hematopoietic cells [2].

In the past few decades, chemotherapy has remained to be the primary treating strategy for most leukemic patients. The rate of complete remission (CR), achieved by modern intensive chemotherapy, is 80–90% in adult patients with acute lymphoblastic leukemia. However, the relapse rate is more than 60% [3]. For these relapsed cases, treatment choices are limited, partially due to drug resistance and decreased tolerance to further chemotherapy [4]. Moreover, hematopoiesis of these patients has also been impaired. Considering the findings of normal hematopoiesis in leukemic hosts mentioned above, we propose that the chemotherapy has impaired hematopoietic regenerative capacity in leukemic patients.

However, quantitative, especially functional changes of primitive hematopoietic and leukemic cells post chemotherapy have not been well clarified yet. In the present study, kinetics of both hematopoietic primitive cells and leukemia cells and functional status of L^−^K^+^S^+^ hematopoietic cells in bone marrow in a treated leukemic mouse model were investigated. In addition, SA-β gal status and gene expression such as *p*16 and EGR1 of bone marrow L^−^K^+^S^+^ hematopoietic cells were tested to obtain more insight in underlying the mechanism. Our data indicated that bone marrow primitive hematopoietic cells regenerated earlier than leukemic cells in a leukemia therapy model, and their repopulating capacity gradually diminished after treatment partly due to senescence caused by accelerated cycling.

## Methods

### Chemotherapeutic agents

Chemotherapeutic agents were Cyclophosphamide (CTX, Jiangsu Hengrui Medicine, CO., LTD) and Cytarabine (Ara-C, Pfizer Italia s.r.l); both were dissolved in PBS at 50 mg/mL and stored at −20°C.

### Determination of maximum tolerated dose (MTD)

MTD of therapeutic agent was defined as the maximum dose causing no death and no more than 10% weight loss. This was determined by treating wide-type, 8-week-old female C57/BL6 J mice (n = 5). Drugs were dissolved in 400 μL PBS and injected intraperitoneal into these mice. Tested doses administered daily for four consecutive days were 200, 100, 50, and 25 mg/kg for CTX, and 200, 150, 100, and 50 mg/kg for Ara-C. Finally, MTD gained was 100 mg/kg for CTX and 150 mg/kg for Ara-C. These doses were then used for primary chemotherapy testing in leukemic mice.

### Mice and cells

C57/BL6 J mice (CD45.2^+^, 8-week-old) were used as leukemic hosts. The T-ALL cells were derived from bone marrow CD45.1^+^Lin^−^ hematopoietic cells of B6.SJL mice, induced by Notch-1 ICN-GFP over-expression, and kindly offered by the State Key Laboratory of Experimental Hematology Tianjin, China. With no pretreatment, each C57/BL6 J mouse was injected with 10^5^ congenic T-ALL cells through tail-vein to induce T-ALL development in 10 days. Wild type C57/BL6 J mice injected with equal volume of PBS were used as normal control. Among the normal control mice, those that received 1-day chemotherapy at MTD doses were defined as the drug-only group. T-ALL mice that received chemotherapy at MTD doses were defined as treated leukemic mice, and sub-grouped as follows: the 1-day treated group, 2-day treated group, 3-day treated group, and 4-day treated group. T-ALL mice received 1-day therapy of half MTD doses were defined as the lower-dose group. T-ALL mice without other interventions were defined as leukemia group.

B6.SJL mice (CD45.1^+^, 8-week-old) were used as recipients and source of competitive cells in the competitive bone marrow transplantation (c-BMT) assays.

All mice were bred and maintained under defined flora according to guidelines established and approved by the Institutional Animal Committees at the State Key Laboratory of Experimental Hematology Tianjin, China.

### Flow cytometry analysis

Murine bone marrow cells were obtained by flushing iliums, femurs and tibias with PBS or PBE. Immuno-phenotypes were used as follows: for murine hematopoietic stem cell (HSC) was Lin^−^c-Kit^+^Sca-1^+^ (LK^+^S^+^), including long-term hematopoietic repopulating HSC (LT-HSC; CD34^−^Flk2^−^LK^+^S^+^), short-term repopulating HSC (ST-HSC; CD34^+^Flk2^−^LK^+^S^+^), and multi-potent progenitor (MPP; CD34^+^Flk2^+^LK^+^S^+^); for murine hematopoietic progenitor cell (HPC) was Lin^−^c-Kit^+^Sca-1^−^ (LK^+^S^−^), sub-divided as granulocyte/macrophage progenitor (GMP; CD34^+^CD16/32^+^LK^+^S^−^), common myeloid progenitor (CMP; CD34^+^CD16/32^−^LKS^−^), and megakaryocyte/erythroid progenitor (MEP: CD34^−^CD16/32^−^LK^+^S^−^). Normal hematopoietic and leukemic cells were discriminated by CD45.2, GFP, or CD45.1 expressions. For detection of HSC/HPC and their sub-populations, we used FITC conjugated CD34 (RAM34, e-Bioscience), APC-cy7 conjugated with a mixture of lineage antibodies (anti-CD3 145-2C11, CD4 GK1.5, CD8 53-6.7, Mac-1 M1/70, B220 RA3-6B2, Gr-1 RB6-8C5, Ter-119 TER-119; all were purchased from e-Bioscience), Streptavidin APC-cy7 (BD), PE-cy7 conjugated Sca-1 (D7, e-Bioscience), APC conjugated c-Kit (2B8, e-Bioscience), PE conjugated CD16/32 (93, e-Bioscience) and Flk2 (A2F10.1, BD), Percp-cy5.5 conjugated CD45.2 (104, BD) and PE conjugated CD45.2 (104, e-Bioscience). All analysis was performed on an LSR Aria II flow cytometer (BD Bioscience) and further studied by FlowJo 7.6 software (FlowJo LLC, Ashland, OR, USA).

### Analysis of apoptosis by flow cytometry

For apoptosis assays, the staining was performed in the staining buffer with FITC conjugated Annexin-V and 7-AAD (5 µL in 100 µL cells for both dyes; BD Pharmigen™ FITC Annexin V Apoptosis Detection Kit) at 37°C for 15 min according to the user’s manual and analyzed using flow cytometry within an hour after staining was completed.

### Cell cycle analysis

For cell-cycle analysis, 100 µL pre-stained cells were fixed, and then after permeabilization, they were further stained with 5 µL PE conjugated Ki-67 (BD) at 37°C for 30 min, then 5 µg Hoechst 33,342 (10 µg/mL, Life Technologies) was added into 500 µL cell suspension before flow cytometry analysis. Cells were discriminated in G0 (Hochest^low^ Ki-67^low^), G1 (Hochest^low^ Ki-67^high^), and G2-S-M (incorporation of both Hochest and Ki-67).

### In vitro colony-forming cell (CFC) assay

CD45.2^+^GFP^−^ BM cells of the leukemic and the 1-day treated groups were sorted on the 1st, 2nd, 5th, and 12th days post chemotherapy for in vitro colony-forming cell assay, respectively. Cells were seeded in methylcellulose medium M3434 (Stem Cell Technologies) and plated in 24-well plates with a 0.5 mL volume at a density of 2 × 10^4^/mL; five replicates per well. Cells were cultured at 37°C, with 5% CO_2_ and ≥95% humidity. After 10 days of culture, colonies were counted under an inverted microscope and recorded in specific lineages.

### Competitive bone marrow transplantation assay

CD45.2^+^ BM cells were sorted from the 1-day treated leukemic mice on the 1st, 2nd, 5th, and 12th days post therapy for c-BMT assay, respectively. A total number of 5 × 10^5^ sorted viable CD45.2^+^ cells together with an equal number of viable CD45.1^+^ competitive cells (from wide-type untreated 8-week-old B6.SJL female mice) were co-transplanted into lethally irradiated (9.5 Gy) female B6/SJL mice (n = 9/group, 8-week-old) through tail-vein injection 6 h after irradiation. After transplantation, tail-vein blood was tested for donor contribution and lineage differentiation 1 month later and monthly for four consecutive months since. Relative contributions of tested (CD45.2^+^) and competitive cells (CD45.1^+^) were analyzed using FITC conjugated CD45.2 (104, Bio-legend) and Percp-cy5.5 conjugated CD45.1 (A20, BD). Differentiation status was analyzed using following lineage markers: APC conjugated Mac-1 (M1/70, e-Bioscience) for myeloid lineage, PE conjugated CD3 (145-2C11, e-Bioscience) for T lineage and PE-cy7 conjugated B220 (RA3-6B2, e-Bioscience) for B lineage. Analysis was done by flow cytometry.

### Senescence analysis using flow cytometry

Senescent status of cells was examined according to the manufacture’s instruction of the ImaGene Green™ C_12_-FDG lacZ Gene Expression Kit (Molecular Probes, Inc.), and further guided by a Nature Protocol suggested method [5].

### Cell sorting procedures

For HSC (LK^+^S^+^ cells) isolation, BM cells were firstly enriched for c-Kit expression by immuno-selection with CD117 conjugated micro-magnetic beads (Miltenyi Biotec) according to the manufacturer’s instructions. Enriched cells were then stained with PE-cy7 conjugated with a mixture of lineage antibodies (anti-CD3 145-2C11, Mac-1 M1/70, Gr-1 RB6-8C5, CD4 GK1.5, B220 RA3-6B2, CD8 53-6.7, Ter-119 TER119; all were purchased from e-Bioscience), PE conjugated Sca-1 (D7, e-Bioscience), APC conjugated c-Kit (2B8, e-Bioscience) and Percp-cy5.5 conjugated CD45.2 (104, e-Bioscience). Normal hematopoietic and leukemic cells were sorted by CD45.2 and GFP expression, respectively; and 4′,6-diamidino-2-phenylindole (DAPI) was used to exclude dead cells during the sorting procedure.

### Quantitative reverse transcriptase PCR (qRT-PCR)

A total number of 2 × 10^4^ BM CD45.2^+^LK^+^S^+^ cells or GFP^+^ cells were sorted directly into the lysis buffer (Stratagene). Total RNA was extracted with the RNA nano-prep kit according to the manufacturer’s instructions (Stratagene). Reverse transcription was achieved using oligo-dT and M-MLV reverse transcriptase (Ambion). Real-time polymerase chain reaction (PCR) was performed with SYBR green Master Mix (Finnzymes), using a Real-time Quantitative PCR 7500 (ABI) machine. Parameters were as follows: Holding stage: 95°C, 10 min, 1 cycle; Cycling stage: 95°C, 15 s, 60°C, 50 s, for 60 cycles; Melt curve stage: 95°C, 15 s, 1 cycle, 60°C, 1 min, 1 cycle, 95°C, 15 s, for 1 cycle. The sequences of all primers used in the qRT-PCR assay are listed in the Additional file 1: Table S1.

### Statistical analysis

Data are presented as mean ± SEM if not indicated otherwise. Survival status was analyzed using Kaplan–Meier analysis. Differences between two groups were analyzed using a two-tail unpaired Student t test. For comparison of multiple groups, one-way ANOVA was used and followed by Dunnett analysis between each of the two groups. Differences with a *P* value ≤0.05 were considered statistically significant.

## Results

### Development of a system for evaluation of chemotherapy on leukemia mice

**Figure 1:**
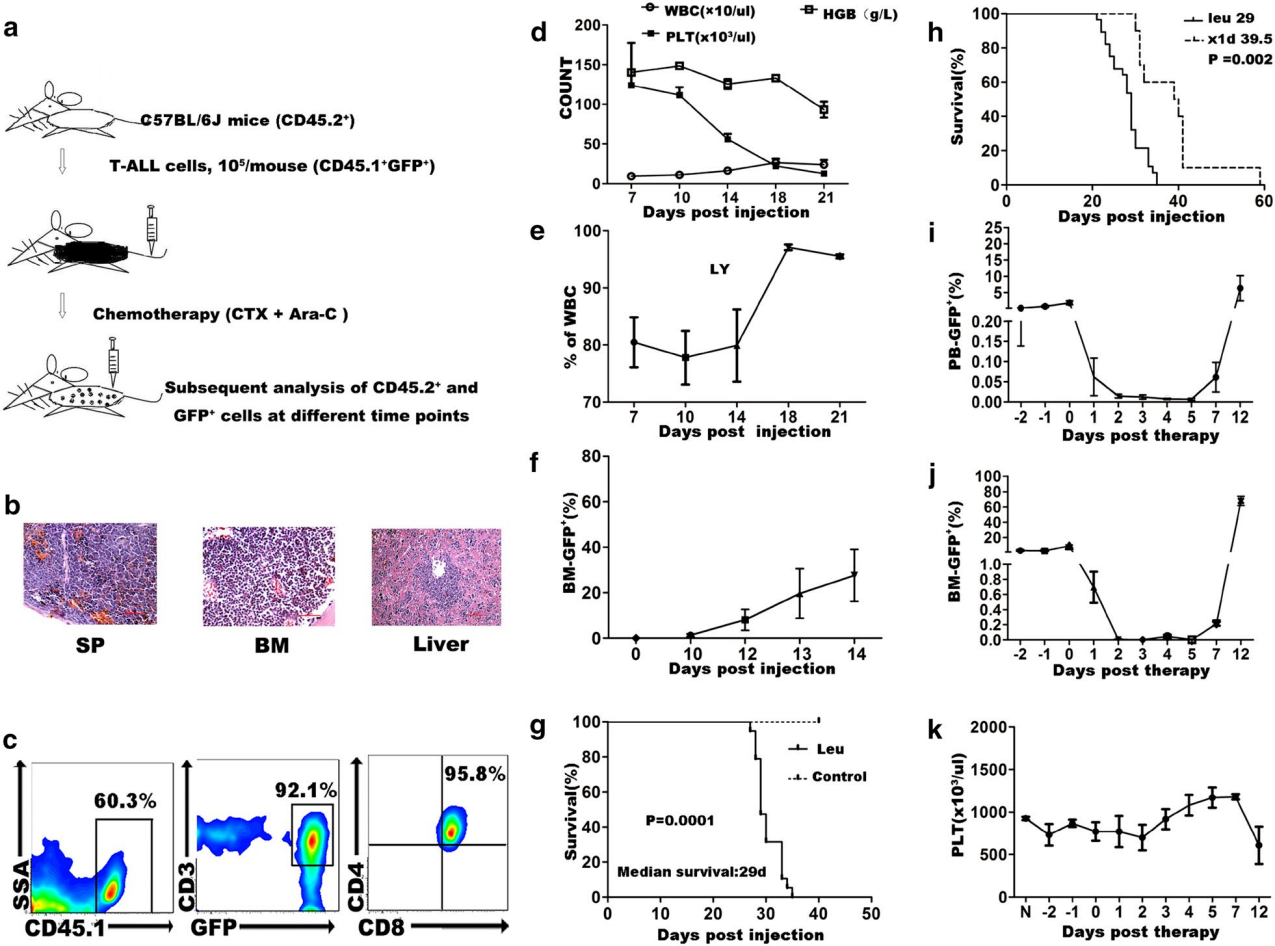
Experimental design and a CR-to-relapse treated leukemic model established in non-irradiated T-ALL mice. **a** Schematic representation of experimental strategy. Eight-week-old C57/BL6 J mice (CD45.2^+^), each injected with 10^5^ congenic T-ALL cells (CD45.1^+^), are used to develop T-ALL. When leukemic cells reach 1–5% of the mononuclear cell (MNC) in peripheral blood (PB) of these mice, chemotherapy (Ara-C and CTX) is given, and both leukemic cells and normal hematopoiesis are examined thereafter. **b** Leukemic infiltration in spleen, bone marrow and liver of the dying T-ALL mice in the late disease stage. Mice injected with T-ALL cells were sacrificed on the 21st day post injection for histopathological evaluation. Tissues were collected and fixed with 10% formalin overnight, stained with hematoxylin-eosin (H&E) and examined by an inverted microscopy. **c** Expression of T-ALL cell markers on flow cytometry for the leukemic cells as GFP^+^CD45.1^+^CD3^+^CD4^+^CD8^+^. **d**–**e** Peripheral blood cell counts of T-ALL mice showed a gradual appearance of lymphocytic leukocytosis and decreased hemoglobin and platelets (n = 3–4). Peripheral blood samples were tested by an automatic whole-blood analyzer (XT-2000T; SysmHGBex). **f** Leukemic burden in BM of T-ALL mice increased daily, and reached nearly 10% of MNC on the 12th day post injection (n = 4). **g** T-ALL mice showed a much shorter life-span compared to the normal control (median survival days post T-ALL cells injection: 29; n = 19; p = 0.0001). **h**–**k** When leukemic burden of the T-ALL mice reached about 1–5% in PB, mice received 1-day therapy composed of Ara-C (150 mg/kg) and CTX (100 mg/kg). **h** The treated leukemic mice (1-day treated leukemic group) showed prolonged survival time compared to the untreated leukemic group (median survival days post T-ALL cells injection: 39.5 vs. 29; n = 10; p = 0.002). **i** Leukemic burden in PB of the treated leukemic mice at different time points post therapy (n = 6–8). **j** Leukemic burden in BM of the treated leukemic mice at different time points post therapy (n = 3–4). Leukemic burden in PB and BM both decreased right after treatment, and regenerated since the 5th day post therapy. It was nearly undetectable on the 2nd to the 5th day post therapy. **k** Platelet count in PB of the treated leukemic mice post therapy (n = 6–9). It showed no significant difference when compared with the PBS injected control mice, though there was a decrease tread on the 12th day post therapy when leukemia relapsed. All data were presented as mean ± SEM.

In order to get insight into the effects of chemotherapy on primitive hematopoietic cells and leukemic cells, we established a leukemia-therapy model as illustrated in Figure 1a. Histopathological examination of dying mice revealed leukemic infiltration in spleen, bone marrow, and liver (Figure 1b). Flow cytometric analysis of leukemic cells confirmed their immunophenotype as CD45.1^+^GFP^+^CD3^+^CD4^+^CD8^+^, indicating T-ALL (Figure 1c). Whole blood cell counts in peripheral blood of these mice showed a gradual decrease of hemoglobin and platelet together with leukocytosis (Figure 1d), as well as an increase of lymphocytes (Figure 1e). Leukemic burden in bone marrow and found it gradually increased (Figure 1f). The leukemic mice had much shorter life-span (median survival time: 29 days; control: no mice died within the 40 inspecting days; *P* = 0.0001; Figure 1g).

With different treat modalities, we set up course-dependent treating models leading to different kinetics of leukemic cells and normal hematopoietic cells (Additional file 2: Figure S1). In this study, we use one-dose treatment model which facilitates the study.

Treatment was administrated when leukemic burden reached 1–5% of mononuclear cells in peripheral blood (Figure 1a). The chemotherapy combination included two drugs, CTX (100 mg/kg) and Ara-C (150 mg/kg) with one dose. The treated leukemic mice lived much longer than those without treatment (median survival days: 39.5 vs. 29 days, *P* = 0.002; Figure 1h). Leukemic cells in peripheral blood and bone marrow decreased rightly post treatment, and reached plateau during day 2–5, then recovered at day 7 (Figure 1i–j). The leukemia cells infiltrated the bone marrow and spleen. (Additional file 3: Figure S2). Of note, we observed a period (the day 2 to the day 5 post therapy) when leukemic cells could hardly be detected with a nearly uncompromised platelet count (Figure 1i–k). We defined this status as complete remission (CR). On day 7, leukemia cells (GFP^+^) relapsed. Thus, we successfully established a CR-to-relapse T cell leukemia model in non-irradiated mice with combined chemotherapy.

### Primitive hematopoietic cells showed an earlier regeneration than leukemic cells

**Figure 2:**
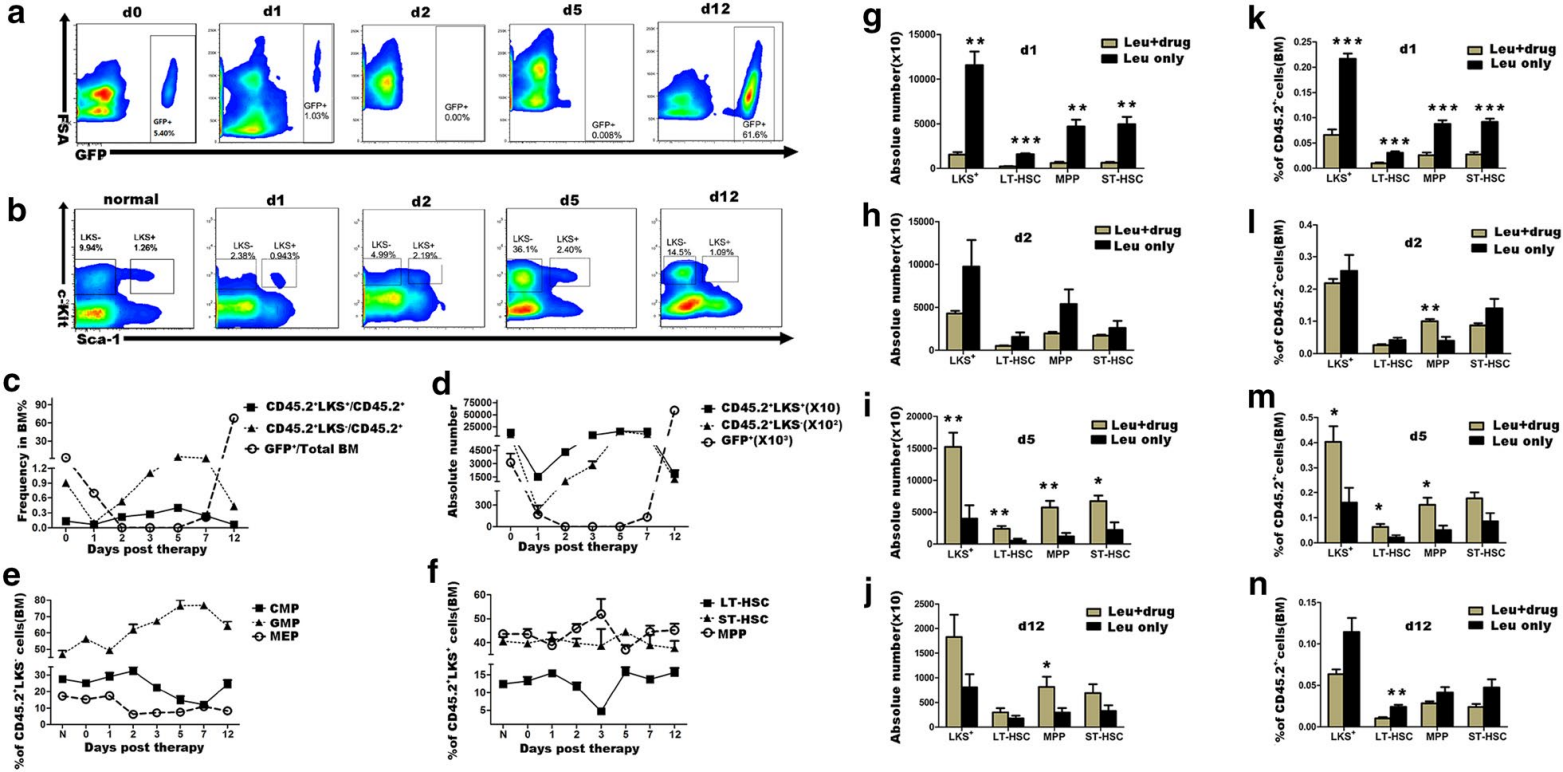
Primitive hematopoietic cells showed an earlier regeneration than leukemic cells post therapy. **a** Representative flow cytometry plots of leukemic cells in the BM of the 1-day treated leukemic mice on the therapeutic day (d0), and on the 1st, 2nd, 5th, and 12th day post therapy. **b** Representative flow cytometry plots of CD45.2^+^LK^+^S^+^ and CD45.2^+^LK^+^S^−^ cells in the BM of normal control (shown as N) and the 1-day treated leukemic mice post therapy. Cells were gated in CD45.2^+^Lin^−^ populations. **c** Frequencies of leukemic cells in whole MNCs and CD45.2^+^LK^+^S^−^ and CD45.2^+^LK^+^S^+^ cells in CD45.2^+^ hematopoietic cell fractions in the BM of the 1-day treated leukemic mice on the therapeutic day (d0) and following days (n = 3–4). **d** Total numbers of leukemic cells, CD45.2^+^LK^+^S^−^ and CD45.2^+^LK^+^S^+^ cells in the BM (double hindlimbs) of the 1-day treated leukemic mice on different time (n = 3–4). **e** Frequencies of CMP, GMP, and MEP in CD45.2^+^LK^+^S^−^ cells in the BM of normal control and the 1-day treated leukemic mice on different time (n = 3–8). **f** Frequencies of LT-HSC, ST-HSC, and MPP in CD45.2^+^LK^+^S^+^ cells in the BM of normal control and the 1-day treated leukemic mice on different time (n = 3–9). **g**–**j** Total numbers of LT-HSC, ST-HSC, MPP, and CD45.2^+^LK^+^S^+^ cells of the leukemia-only control and the 1-day treated leukemic mice in BM (double hindlimbs) on different days (n = 3–6). **k**–**n** Frequencies of LT-HSC, ST-HSC, MPP and CD45.2^+^LK^+^S^+^ cells in CD45.2^+^ hematopoietic cell fractions in the BM of the leukemia-only control and the 1-day treated leukemic mice on different days (n = 3–6). All data were presented as mean ± SEM. Statistical significance determined as: *p < 0.05; **p < 0.01; ***p < 0.001.

An important characteristic of HSC is its self-renewal ability, which is key for its quantity and quality maintenance [6]. We quantitatively analyzed bone marrow primitive hematopoietic cells in the one-dose treated leukemia model. Both CD45.2^+^L^−^K^+^S^+^, CD45.2^+^L^−^K^+^S^−^ hematopoietic cells and their sub-populations were analyzed by flow cytometry. CD45.2^+^L^−^K^+^S^+^ hematopoietic cells decreased on day 1 post therapy (mean number: 1.523 ± 271.4 × 10^4^), and regenerated rapidly from day 2 (mean number: 4.298 ± 2.866 × 10^4^). And the cell number reached the peak on the 5th day, and gradually decreased since (mean number: 15.217 ± 2.243 × 10^4^) on the 5th day; 14.960 ± 0.7069 on the 7th day; 1.825 ± 0.4581 × 10^4^ on the 12th day). CD45.2^+^L^−^K^+^S^−^ hematopoietic cells showed a similar trend. However, leukemic cells began to regenerate since the 5th day and continued proliferating thereafter (Figure 2c, d, Additional file 4: Figure S3A, B).

We then analyzed sub-populations of CD45.2^+^L^−^K^+^S^−^ and CD45.2^+^L^−^K^+^S^−^ hematopoietic cells in bone marrow. We found that since the recovery phase, CD45.2^+^L^−^K^+^S^−^ hematopoietic cells showed a granulocyte monocyte progenitor (GMP)-biased manner when compared with control (on the 2nd day 61.80 ± 3.25%; on the 3rd day 67.18 ± 1.93%; on the 5th day 76.50 ± 3.46%; on the 7th day 76.75 ± 1.63%; on the 12th day 64.00 ± 2.84%; all *P* values <0.05 when compared with control; Figure 2e). We had similar results for colony-forming cell assays (Additional file 5: Figure S4B–E). For CD45.2^+^L^−^K^+^S^−^ hematopoietic cells, on the 3rd day CD45.2^+^LK^+^S^+^ hematopoietic cells showed a decreased frequency of phenotypically defined LT-HSC compared to control (4.73 ± 0.61% vs. normal 12.44 ± 0.69%, *P* < 0.0001; Figure 2f). This might be a compensating loss of mature cells caused by leukemia and chemotherapy; since the 5th day post therapy, frequencies restored back to a nearly normal level.

We then compared the one-dose treated leukemic mice with the untreated leukemia group in the late leukemia relapsing stage (on the 12th day). Although there was no significant difference in absolute numbers (296.2 ± 87.88 vs. 174.0 ± 53.93, *P* = 0.252; Figure 2j), CD45.2^+^L^−^K^+^S^−^ hematopoietic cells showed a significant lower percentage in treated group (0.01 ± 0.001% vs. 0.024 ± 0.003%, *P* = 0.008; Figure 2n), suggesting that chemotherapy leading to a decreased frequency of LT-HSC.

We also tested related parameters in the lower-dose, the four-dose treated and the treatment only groups. In the lower-dose treated group, we observed a similar trend of primitive hematopoietic cells and leukemic cells, although to a less extent (Additional file 6: Figure S5). In the four-dose treatment group, we found that leukemic burden in peripheral blood was continuously undetectable within 90 days post therapy, and primitive hematopoietic cells in bone marrow could recover back to normal status post therapy (Additional files 2, 7: Figures S1, S6). Moreover, CD45.2^+^LK^+^S^−^ cells showed a same shift towards GMP differentiation comparable to the one-dose treated leukemic group during the early recovery phase (Additional file 7: Figure S6). We also tested these in the treatment only group. Data showed that the frequencies of both L^−^K^+^S^+^ and L^−^K^+^S^−^ cells recovered to the normal in the end (Additional file 8: Figure S7A–C).

In the present model, primitive hematopoietic cells and leukemic cells showed different kinetics together with a GMP-bias of the hematopoietic progenitors and an additional consumption of phenotypically defined LT-HSC possibly due to chemotherapy.

### Transient and intensive proliferation of bone marrow L^−^K^+^S^+^ hematopoietic cells

**Figure 3:**
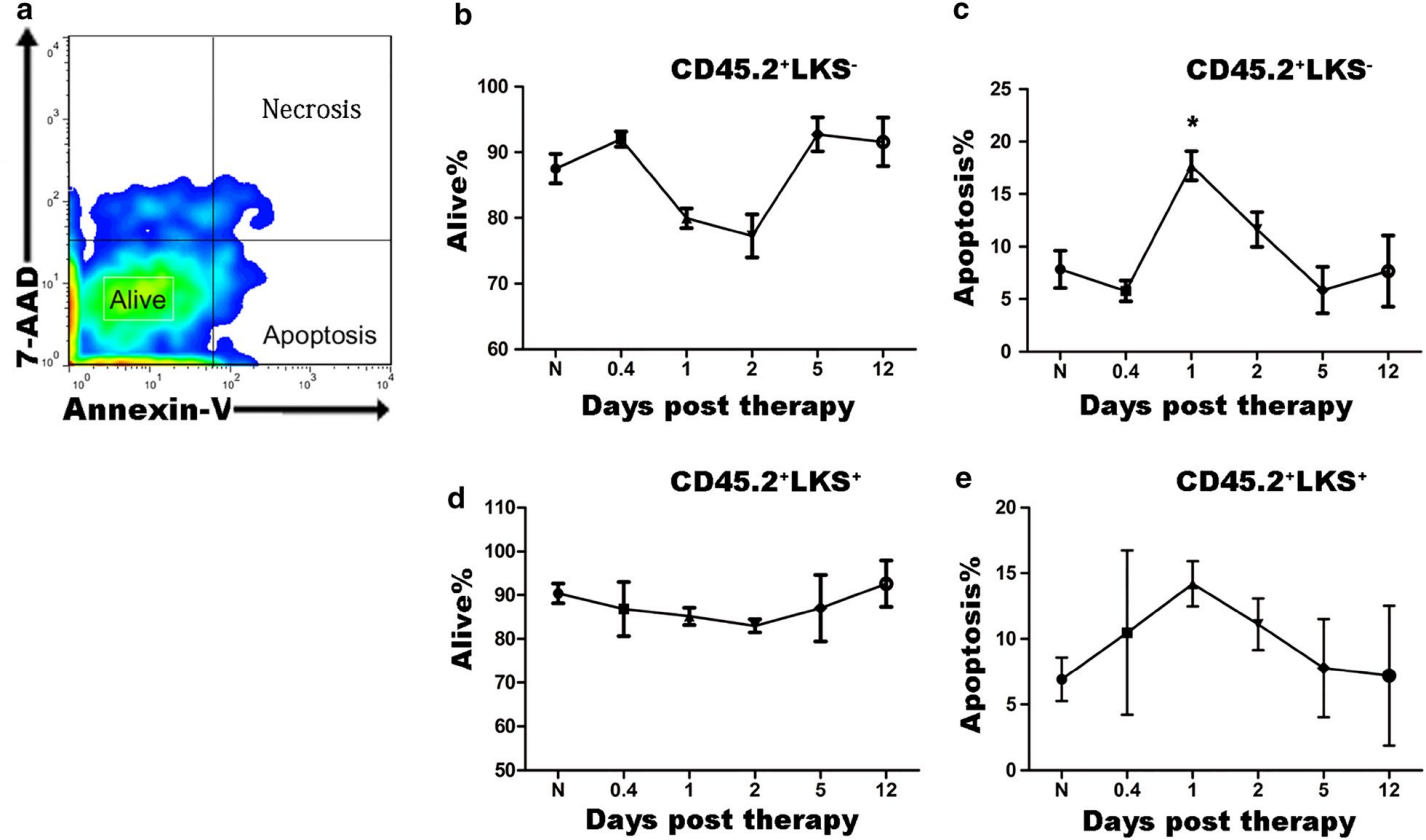
Apoptosis has little effect on changes of LK^+^S^−^/LK^+^S^+^ hematopoietic cells since the recovery phase. **a** Gating strategy for apoptosis using 7-AAD and Annexin-V staining. The cellular uptake of these dyes discriminated cells in Alive (7-AAD^low^ Annexin-V^low^), Necrosis (Annexin-V^low^ 7-AAD^high^) and Apoptosis (Annexin-V^high^ 7-AAD^low^). **b**–**e** Percentages of viable CD45.2^+^LK^+^S^−^ cells (**b**), apoptotic CD45.2^+^LK^+^S^−^ cells (**c**), viable CD45.2^+^LK^+^S^+^ cells (**d**), apoptotic CD45.2^+^LK^+^S^+^ cells (**e**) in the BM of normal control (shown as N) and the 1-day treated leukemic mice on different days (n = 4–5).

Cell proliferation and apoptosis are two opposite factors influencing cell numbers. Next, we further investigated these parameters in L^−^K^+^S^−^ and L^−^K^+^S^+^ hematopoietic cells. We chose the 1st, 2nd, 5th, and 12th days post therapy as further testing time-points in the one-dose treated leukemic group and results were compared with the control group. Nine hours after therapy (presented as 0.4 day) was also tested to exclude the possibility of missing early acting phase. Compared with the control, although with no statistical significance, treated leukemic mice showed increased percentages of apoptosis of CD45.2^+^L^−^K^+^S^−^ cells early after therapy, while no differences thereafter (day 0: 7.85 ± 1.78%; 9 h: 5.78 ± 1.00%; day 1: 17.70 ± 1.39%; day 2: 11.64 ± 1.65%; day 5: 5.87 ± 2.23%; day 12: 7.67 ± 3.39; all *P* values >0.05 when compared to normal except day 1, Figure 3). Data showed that apoptosis was involved in the decrease of primitive hematopoietic cells post therapy, especially in the early phase.

**Figure 4:**
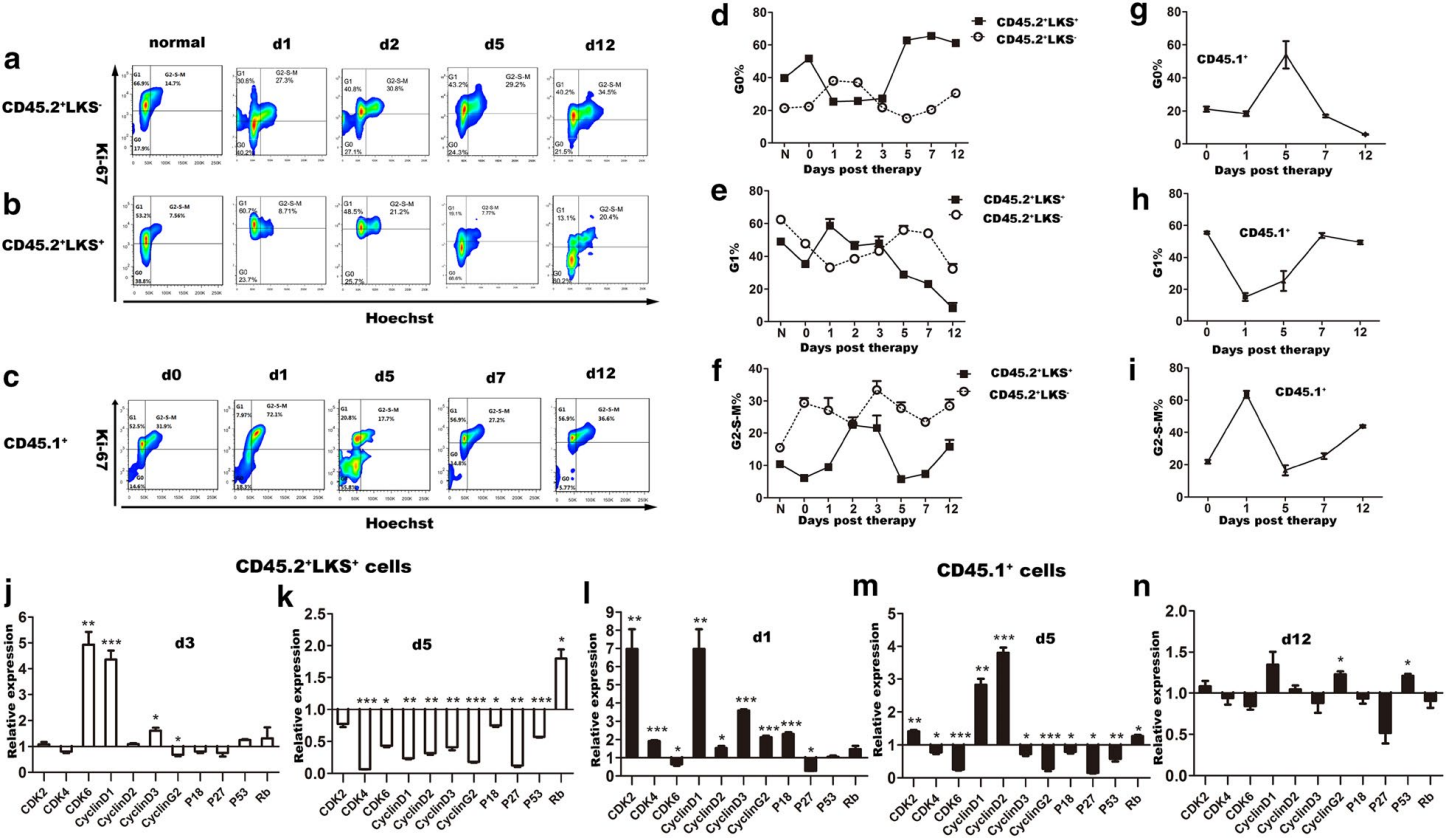
Primitive hematopoietic cells showed a dramatic cell-cycle activation after therapy ahead of leukemic cells. Cell cycle status of primitive hematopoietic cells and leukemic cells in the BM post therapy was analyzed by Ki-67 and Hoechst staining on flow cytometry. CD45.2^+^LK^+^S^+^ cells and leukemic cells post therapy were sorted and the expression patterns of cell-cycle related genes in these cells were examined. CD45.2^+^LK^+^S^+^ cells from normal control and leukemic cells sorted from leukemia-only mice were used as control for either cell type, respectively. **a** Representative flow cytometry plots of cell-cycle of CD45.2^+^LK^+^S^−^ cells in the BM of normal control and the 1-day treated leukemic mice on different days as indicated. **b** Representative flow cytometry plots of cell-cycle of CD45.2^+^LK^+^S^+^ cells in the BM of normal control and the 1-day treated leukemic mice on different days. **c** Representative flow cytometry plots of cell-cycle of leukemic cells in the BM of the 1-day treated leukemic mice on different days. **d**–**f** Cell-cycle status of CD45.2^+^LK^+^S^−^ and CD45.2^+^LK^+^S^+^ cells in the BM of normal control and the 1-day treated leukemic mice on different days (n = 3–7). **g**–**i** Cell-cycle status of leukemic cells in the BM of the 1-day treated leukemic mice on different days (*n* = 4–8). **j**–**k** Gene expression patterns of CD45.2^+^LK^+^S^+^ cells in the BM of normal control and the 1-day treated leukemic mice on the 3rd and 5th day post therapy. **l**–**n** Gene expression patterns of leukemic cells in the BM of the leukemia-only and the 1-day treated leukemic mice on the 1st, 5th, and 12th day post therapy. All data were presented as mean ± SEM. Statistical significance determined as: *p < 0.05; **p < 0.01; ***p < 0.001.

Then we examined cell cycle status of both primitive hematopoietic cells and leukemic cells in bone marrow of the 1-day treated leukemic mice. Figure 4a–c showed the representative flow cytometry plots for the CD45.2^+^LK^+^S^−^, CD45.2^+^LK^+^S^+^ hematopoietic cells, and CD45.1^+^ leukemic cells, while statistical analyses are presented in Figure 4d–i. CD45.2^+^LK^+^S^−^ hematopoietic cells exhibited a relatively stable status, a much larger part of these cells kept in G2-S-M phase compared to normal control, indicating a much more active proliferation status of these cells post therapy (Figure 4d–f). While for CD45.2^+^LK^+^S^+^ hematopoietic cells, they went through complex changes of cell cycle. A large proportion of these cells rapidly left G0 phase and entered G2-S-M proliferating period post therapy (mean percentage of cells in G2-S-M phase %: on the therapeutic day 6.11 ± 0.63; on the 1st day post therapy 9.48 ± 1.06; on the 2nd day 22.55 ± 0.64; Figure 4f). As expected, when leukemia relapsed, they went back into arrest (mean percentage of cells in G2-S-M phase on the 5th day: 5.79 ± 0.86%; Figure 4f). However, in the late leukemia relapsing stage, we found that there was a large percentage of CD45.2^+^LK^+^S^+^ hematopoietic cells in G2-S-M phase compared with normal control (mean percentage of cells in G2-S-M phase %: on the 12th day 15.78 ± 2.11 vs. normal 10.37 ± 0.98; p = 0.026; Figure 4f). However, in the drug-only group, cell cycle status of these cells returned to normal in the end though they also experienced complex changes during the hematopoietic recovery phase (Additional file 8: Figure S7D–F). When we focused on leukemic cells in bone marrow of the leukemia-therapy mice, we found they entered proliferation later than CD45.2^+^LK^+^S^+^ hematopoietic cells. They showed up again since the 5th day, and entered into a persistent proliferation period since the 7th day post therapy. Although frequencies of leukemic cells in G0 phase decreased, G2-S-M showed a relatively stable status except for the 1st day post therapy. Interestingly, a large proportion of leukemic cells was in G2-S-M phase, together with significantly decreased G1 proportion on the 1st day post therapy compared to the therapeutic day (G2-S-M phase frequency %: 63.54 ± 2.16 vs. 21.83 ± 1.13, p < 0.0001; G1 phase frequency: 15.21 ± 2.49 vs. 55.5 ± 0.74, p < 0.0001; G0 phase frequency: 18.35 ± 1.45 vs. 21.18 ± 1.55, p = 0.256; Figure 4g–i). These results were confirmed in the lower-dose treated group (Additional file 6: Figure S5). Gene analysis found coincident expression changes of cell cycle related genes, and CDK4, CDK6, and Cyclin D1 were the main different ones, indicating their possible role in mediating different changes of leukemic cells and CD45.2^+^LK^+^S^+^ hematopoietic cells post therapy (Figure 4j–n).

Data presented above indicate that cell proliferation and apoptosis were both presented in a dynamic manner post therapy. Cell cycling was the main factor responsible for different changes of primitive hematopoietic cells and leukemic cells post therapy; while apoptosis mainly affected the early phase post therapy, and was apparently ignorable since recovery of both CD45.2^+^LK^+^S^+^ and CD45.2^+^LK^+^S^−^ hematopoietic cells.

### Gradually diminished regenerative capacity of primitive hematopoietic cells

**Figure 5:**
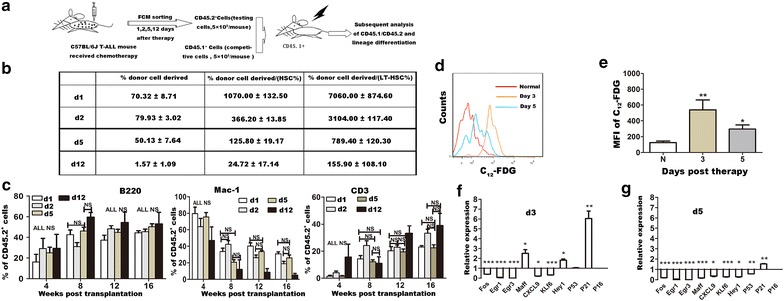
Gradual loss of regenerative capacity of primitive hematopoietic cells involved in the treated leukemic model, caused by excessive proliferation related senescence. **a** Competitive bone marrow transplantation assays were used to examine the regenerative capacity of hematopoietic cells in vivo. CD45.1^+^ bone marrow cells from wild-type B6.SJL female mice (5 × 10^5^) and CD45.2^+^ bone marrow cells (5 × 10^5^) sorted from the 1-day treated leukemic mice on different days post therapy were co-transplanted into each B6.SJL mice (CD45.1^+^) which have undergone lethal irradiation (9.5 Gy). Donor contribution and lineage differentiation status were examined 1 month later at a frequency of once per month for four consecutive months. **b** Donor contribution on the fourth month post transplantation was examined and deeply analyzed combined with the frequencies of HSC (CD45.2^+^LK^+^S^+^ cells) and LT-HSC (CD45.2^+^LK^+^S^+^CD34^−^Flk2^−^ cells) in BM CD45.2^+^ hematopoietic cell fractions of the 1-day treated leukemic mice on the indicated days tested by flow cytometry (n = 3–9). **c** Lineage differentiation status of the 4 months post transplantation (n = 3–9). **d**, **e** Higher levels of senescence confirmed in primitive hematopoietic cells post therapy compared to normal control. Senescence status of CD45.2^+^LK^+^S^+^ cells in the BM of normal control and the 1-day treated leukemic mice were tested on the 3rd and 5th day post therapy by C_12_-FDG staining. The representative flow cytometry plots are shown in **d**, while the statistic results (n = 7–12) are shown in **e**. **f**, **g** Expression patterns of genes regulating HSC function in primitive hematopoietic cells changed post therapy. CD45.2^+^LK^+^S^+^ cells of normal control and the 1-day treated leukemic mice on the 3rd and 5th day post therapy were sorted out and used for analysis of gene expression patterns, including p16, p21, p53, EGR1 and FOS. All data were presented as mean ± SEM. Statistical significance determined as: *p < 0.05; **p < 0.01; ***p < 0.001; *ns* not significant.

Another important feature of HSCs is their ability to reconstitute long-term multi-lineage hematopoiesis [6]. We therefore further examined the regeneration capability with competitive bone marrow transplantation assays using HSCs harvesting on the 1st, 2nd, 5th, and 12th day post therapy. CD45.2^+^ bone marrow hematopoietic cells were sorted out on the indicated days and co-transplanted with equal number of CD45.1^+^ competitive cells into lethally irradiated mice (Figure 5a). The donor contribution of the hematopoietic cells sorted at the four checking-points post therapy gradually decreased, indicating a gradual loss of repopulating ability of bone marrow hematopoietic cells in our model. To exclude influences of the different frequencies of primitive hematopoietic cells in whole bone marrow of the four independent tested cell populations, we further calculated the donor contributions of whole bone marrow hematopoietic cells over the corresponding frequencies of bone marrow primitive hematopoietic cells (herein referred to HSC and LT-HSC). We also observed a gradual loss of repopulating capabilities, providing further evidence of dysfunction of primitive hematopoietic cells in reconstitution. Lineage differentiation showed no difference in the first 2 months, however, in the following 2 months, myeloid differentiation seemed to be impaired (Figure 5c). These results suggested that normal hematopoiesis were impaired in the one-dose treated leukemic hosts as shown by diminished repopulating ability and impaired myeloid differentiation.

### Senescence is confirmed in L^−^K^+^S^+^ hematopoietic cells

Cellular senescence is usually a result of extensive replication, and mainly occurs prematurely after cells are exposed to oncogenic, oxidative, or genotoxic stress [7–9]. Reports have discussed the role of senescence in mediating impairment of HSC function. In the hematopoietic system, studies mainly focused on irradiation [10, 11]. Since HSCs went through accelerated cycling together with decreased regenerative function and atypical differentiation pattern, a high level of cell senescence is therefore expected in these HSCs. Senescent cells express increased levels of biomarkers, such as SA-β gal and P16 [12, 13]. Here we employed a enzymatic method suggested by Nature Protocols to detect SA-β gal activity. The method involves the alkalinization of lysosomes, followed by the use of 5-dodecanoylaminofluorescein di-β-d-galactopyranoside (C_12_FDG), a fluorogenic substrate of SA-β gal which becomes fluorescent after cleaved by the enzyme and can be detected by flow-cytometry [5]. Senescent status of CD45.2^+^L^−^K^+^S^+^ hematopoietic cells in our 1-day treated leukemic model on the 3rd and 5th day post therapy were measured, and compared to the normal control. CD45.2^+^L^−^K^+^S^+^ hematopoietic cells of the 1-day treated leukemic mice on the 3rd and 5th days post therapy showed higher mean fluorescence intensity (MFI) of C_12_-FDG (normal vs. day 3: 124.6 ± 20.27 vs. 539.7 ± 126, p = 0.0069; normal vs. day 5: 124.6 ± 20.27 vs. 296.6 ± 51.94, p = 0.0259; Figure 5e), indicated higher levels of senescence.

We then analyzed gene expressions of *p16*, *p21*, and *p53*, three key genes involved in cellular senescence [14–16]. Data showed higher expression of P16 and P21 in CD45.2^+^LK^+^S^+^ hematopoietic cells on the 3rd and 5th days post therapy, further confirming the result of senescence (Figure 5f–g).

Moreover, we also observed down-regulated expressions of Egr1 and FOS on these 2 days (Figure 5f, g). Egr1 is a gene affecting hematopoiesis. Although loss of Egr1 does not impair regenerative ability in primary recipient mice, Egr1^−/−^ HSCs exhibit premature loss of function during serial transplantation, suggesting its protective function of HSC in the case of replicative stress [17, 18]. FOS is also found to be involved in stem cell maintenance, stem cell niche interactions and oxidative stress response [19, 20]. These results suggest that excessive proliferation induced the functional impairment.

## Discussion

Leukemia patients usually die of refractory disease, relapse and treatment related mortality, so studies focusing on these contexts will be beneficial. Our group in 2009 reported kinetics of primitive hematopoietic cells in an irradiated T-ALL mouse model [1]. In that model, the number of primitive hematopoietic cells decreased but the cell function was reserved, which may provide an explanation on how autologous stem cell transplantation works. In the present study, we showed primitive hematopoietic cells went through a highly proliferating phase after therapy and exhausted in the end, and leukemic cells kept on growing at a much later starting time point (on the 5th day after therapy). Our data showed that primitive hematopoietic cells and leukemic cells showed different proliferation pattern in the leukemia model under chemotherapy stress. To our knowledge, this is the first time to experimentally demonstrate the earlier regeneration of primitive hematopoietic cells than leukemic cell after chemotherapy. Further analysis of cell cycle and survival status showed cell cycle was involved, which might provide potential therapeutic targets for leukemia through cell cycle regulation.

In the irradiated T-ALL mouse model [1], quantity of primitive hematopoietic cells was decreased; however when transplanted into normal hosts, they could successfully reconstitute hematopoiesis comparable to the normal control, indicating that these primitive hematopoietic cells preserved their functionalities in the leukemic context. This suggests that alterations took place in micro-environment rather than in primitive hematopoietic cells themselves. In the present study, we found primitive hematopoietic primitive cells altered both in quantity and in quality. They showed compromised repopulating capability in the competitive bone marrow transplantation assay. In clinical practice, doctors might meet some difficulties in collecting enough primitive hematopoietic cells for autologous stem cell transplantation from patients of multiple myeloma, lymphoma, and leukemia, especially for those who had received high doses or cycles of chemotherapy or therapy including some special agents [21, 22]. As recently reported, primitive hematopoietic cells showed functional injury due to excessive-proliferation and direct toxicity in aged hosts and hosts suffering from chronic infection or acute serious infection [23–27]. Similar mechanism may be applied in our 1-day treated leukemic model. Moreover, another study found loss of quiescence and impaired function of CD34^+^CD38^low^ cells 1 year following autologous stem cell transplantation. They showed the diminished regenerative capacity was possibly due to a loss of quiescence and a reduced tolerance to oxidative stress [20]. Our data may offer insights on the functional impairment of patients who received autologous stem cell transplantation, as all of them would had chemotherapy prior to the transplantation. Further studies are probably needed to re-evaluate the potential toxicity of traditional chemotherapy to primitive hematopoietic cells in future practice.

In the present study, we had some rather interesting findings worth further investigation. The first one was the myeloid-bias (GMP-bias) of L^−^KS^−^ hematopoietic cells observed in the recovery phase, similar to those observed in aged hosts and many other pathological or physiological conditions [23, 24, 27]. Under most of these conditions, primitive hematopoietic cells displayed decreased regenerative ability. Thus, myeloid-bias may be a common phenomenon accompanied with impaired hematopoiesis, and myeloid lineage may have priority in hematopoietic differentiation under stress. Secondly, we found a large part of leukemic cells in G2-S-M phase 1 day post therapy. One report showed that DNA damage of quiescent HSC accumulating during aging was repaired by entering cycling, and was important for their self-maintenance [28]. Leukemic cells may use the same mechanism to recovery from chemotherapeutic attack.

In summary, our study demonstrates different kinetics of leukemic cells and primitive hematopoietic cells via different cell cycling following chemotherapy. These findings may help us to develop more effective therapeutic methods for leukemia through targeting cell cycling. Our data also provide important new insights into the mechanism by which chemotherapy causes impaired hematopoiesis via induction of premature senescence in leukemic patients. A better understanding of these mechanisms will allow us to develop new interventions to circumvent chemotherapy-induced toxicity to BM via targeted inhibition of chemotherapy-induced HSC senescence, and this might further improve the chemotherapeutic tolerance, especially for those relapsing or refractory patients. This will undoubtedly benefit leukemic patients undergoing chemotherapy, especially those intending for autologous stem cell transplantation.

## Conclusion

In leukemic host, primitive hematopoietic cells in bone marrow enter proliferation earlier than leukemic cells after chemotherapy, at the cost of regenerative capacity possibly impaired by senescence due to accelerated cycling. These findings provide insights on the mechanisms of leukemic relapse, as well the potential toxicity of chemotherapy to primitive hematopoietic cells.

## Additional files

Additional file 1:
**Primer sequences.** Sequences of all the primers used in qRT-PCR. Additional file 2:
**Course-dependent chemotherapy response of the T-ALL mice.** When leukemic cells reached 1-5% in PB, mice received chemotherapy composed of CTX (100 mg/kg) and Ara-C (150 mg/kg) for a consecutive 1, 2, 3 and 4 days, respectively. (**A**) Median survival days post leukemic cell injection were 29, 39.5, 45 and 71.5 for the leukemia-only, one-day treated, two-day treated and three-day treated group, respectively (n = 6-10). For the four-day treated group, no mice died within the inspecting 90 days (n = 6). (**B**) Leukemic burden in PB of the four differently treating groups (n = 6). Data showed that longer the therapeutic course, longer the relapse-free period. The day on which leukemic cells showed up again in PB for the one-day, two-day and three-day treated groups were the 8th, 11th and 17th day post therapy, respectively. While for the four-day treated group, no appearance of relapse was detected within the inspecting 90 days. (**C**) White blood cell count in PB of the four differently treating groups (n = 6). Data showed that longer the therapeutic course, heavier the depression of white blood cells post therapy. (**D**) Platelet count in PB of the four differently treating groups (n = 6). Data showed that longer the therapeutic course, longer the suppression period of platelet in PB post therapy. All data were presented as mean±SEM. Statistical significance as: * p<0.05; ** p<0.01; *** p<0.001. Additional file 3:
**Histopathology of the one-day treated leukemic mice.** Mice of the one-day treated leukemic group were sacrificed at different time points post therapy for histopathological analysis. Tissues were gained right after sacrifice, fixed in 10% formalin overnight, stained by hematoxylin-eosin (H&E) and examined by an inverted microscopy. (**A**) Status of leukemic infiltration and normal hematopoiesis in spleen post chemotherapy. (**B**) Status of leukemic infiltration and normal hematopoiesis in bone marrow post therapy. Additional file 4:
**Kinetics of HSPCs and leukemic cells involved in the one-day treated leukemic group.** (**A**) Frequencies of leukemic cells in whole MNCs and CD45.2^+^LK^+^S^+^ and CD45.2^+^LK^+^S^−^ cells in CD45.2^+^ hematopoietic cell fractions in the BM of the one-day treated leukemic mice (n = 3-4). (**B**) Total numbers of leukemic cells, CD45.2^+^LK^+^S^+^ and CD45.2^+^LK^+^S^−^ cells in BM (double hindlimbs) of the one-day treated leukemic mice (n = 3-4). (**C**) Statistic analysis of sub-populations of CD45.2^+^ hematopoietic cell fractions in the one-day treated leukemic mice (n = 3-4). All data were presented as mean ± SEM. Additional file 5:
**Results of the in vitro colony-forming cell assays in the one-day treated leukemic mice.** CD45.2^+^ hematopoietic cells in the BM of the one-day treated leukemic mice were sorted for in vitro colony-forming cell assays on different days post therapy. CD45.2^+^ hematopoietic cells in the BM of leukemia-only mice were used as control. (**A**–**D**) Results of the CFC assays (n = 4-5). Data showed similar changing trend of hematopoietic progenitors and the same granulocyte shift in the proliferation phase coincident with flow cytometric data. All data were presented as mean±SEM. Statistical significance as: * p<0.05; ** p<0.01; *** p<0.001. Additional file 6:
**Kinetics of HSPCs and leukemic cells involved in the lower-dose treated leukemic mice.** When leukemic cells reached 1-5% in PB, mice received one-day of lower-dose therapy, composed of 75 mg/kg Ara-C plus 50mg/kg CTX. Then changes of leukemic cells and primitive hematopoietic cell fractions were tested. (**A**) Frequencies of leukemic cells in whole MNCs and CD45.2^+^LK^+^S^−^, CD45.2^+^LKS^+^ cells in CD45.2^+^ hematopoietic cell fractions in the BM of the lower-dose treated leukemic mice (n = 3-4). (**B**) Frequencies of LKS^−^ cells, CMP, GMP and MEP in CD45.2^+^ hematopoietic cell fractions in the BM of normal control and the lower-dose treated leukemic mice on the 1st, 2nd and 3rd day post therapy (n = 5-9). (**C**) Frequencies of LK^+^S^+^ cells, LT-HSC, MPP and ST-HSC in CD45.2^+^ hematopoietic cell fractions in the BM of normal control and the lower-dose treated leukemic mice on different days post therapy (n = 5-9). (**D**–**F**) Cell-cycle status of leukemic cells, CD45.2^+^LK^+^S^−^ and CD45.2^+^LK^+^S^+^ cells in the BM of the lower-dose treated leukemic mice on the therapeutic day (d0), and on the six consecutive days since (n = 3-5). All data were presented as mean±SEM. Statistical significance as: * p<0.05; ** p<0.01; *** p<0.001. Additional file 7:
**Kinetics of HSPCs and leukemic cells involved in the four-day treated leukemic group.** When leukemic cells reached 1-5% in PB, mice received four consecutive days of treatment, composed of 150 mg/kg Ara-C plus 100 mg/kg CTX daily. Then changes of leukemic cells and primitive hematopoietic cell fractions were tested. (**A**) Leukemic burden in the BM was continuously undetectable within 40 days post therapy (n = 5). (**B**) Bone marrow cellularity showed a gradual recovery post therapy (n = 5). (**C**) Frequencies of LK^+^S^+^ cells, LT-HSC, MPP and ST-HSC in CD45.2^+^ hematopoietic cell fractions in the BM of normal control and the four-day treated leukemic mice post therapy (n = 3-5). (**D**) Total numbers of hematopoietic LK^+^S^+^ cells, LT-HSC, MPP and ST-HSC in the BM (double hindlimbs) of normal control and the four-day treated leukemic mice on different days post therapy (n = 3-5). (**E**) Frequencies of LKS^−^ cells, GMP, MEP and CMP in CD45.2^+^ hematopoietic cell fractions in the BM of normal control and the four-day treated leukemic mice on different days post therapy (n = 3-5). (**F**) Total numbers of hematopoietic LK^+^S^−^ cells, GMP, MEP and CMP in the BM (double hindlimbs) of normal control and the four-day treated leukemic mice on different days post therapy (n = 3-5). All data were presented as mean±SEM. Statistical significance as: * p<0.05; ** p<0.01; *** p<0.001. Additional file 8:
**Kinetics of HSPCs involved in the drug-only group.** Normal C57/BL6J mice were given one-day therapy, composed of 150 mg/kg Ara-C plus 100mg/kg CTX. Then changes of primitive hematopoietic cell fractions were tested. (**A**) Frequencies of CD45.2^+^LK^+^S^+^ and CD45.2^+^LK^+^S^−^ cells in the BM CD45.2^+^ hematopoietic cell fractions of normal control and the drug-only group mice on different days post therapy (n = 3-5). (**B**) Frequencies of LT-HSC, ST-HSC and MPP in the BM CD45.2^+^LK^+^S^+^ hematopoietic cell fractions of normal control and the drug-only group mice on different days post therapy (n = 3-5). (**C**) Frequencies of CMP, GMP and MEP in the BM CD45.2^+^LK^+^S^−^ hematopoietic cell fractions of normal control and the drug-only group mice on different days post therapy (n = 3-5). (**D**) Frequencies of BM CD45.2^+^LK^+^S^+^ and CD45.2^+^LK^+^S^−^ cells in G0 phase of normal control and the drug-only group mice on different days post therapy (n = 3-5). (**E**) Frequencies of BM CD45.2^+^LK^+^S^+^ and CD45.2^+^LK^+^S^−^ cells in G1 phase of normal control and the drug-only group mice on different days post therapy (n = 3-5). (**F**) Frequencies of BM CD45.2^+^LK^+^S^+^ and CD45.2^+^LK^+^S^−^ cells in G2-S-M phase of normal control and the drug-only group mice on different days post therapy (n = 3-5). All data were presented as mean ± SEM. Statistical significance as: * p<0.05; ** p<0.01; *** p<0.001.
