# Supplementary material for: Excessive proliferation and impaired function of primitive hematopoietic cells in bone marrow due to senescence post chemotherapy in a T cell acute lymphoblastic leukemia model
Source: J Transl Med. 2015 Jul 17;13:234. doi: 10.1186/s12967-015-0543-8 (PMC4504405; doi:10.1186/s12967-015-0543-8)
Supplement: Additional file 1: — Primer sequences. Sequences of all the primers used in qRT-PCR. [file 12967_2015_543_MOESM1_ESM.ppt]

## Slide 1
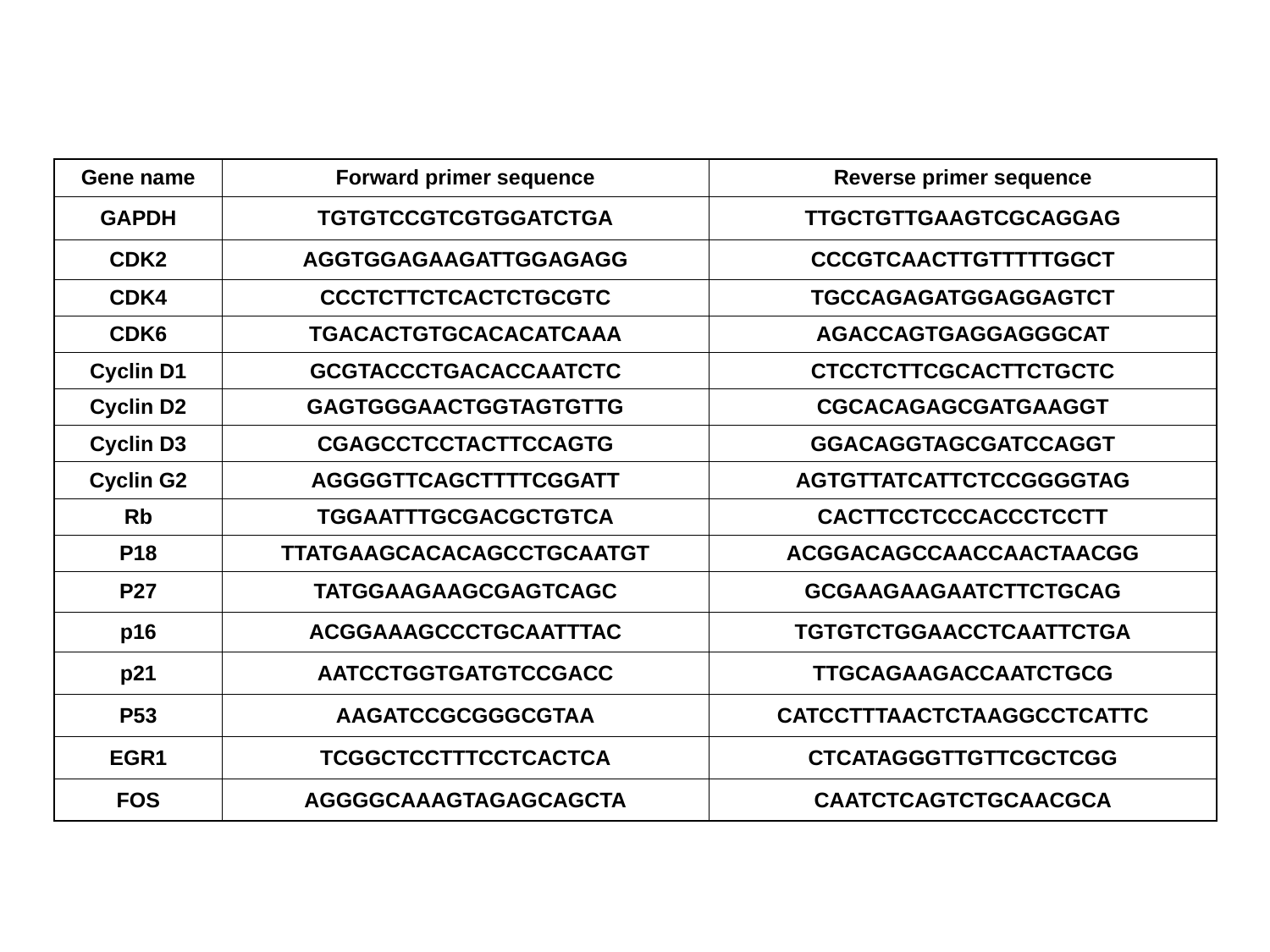

| Gene name | Forward primer sequence | Reverse primer sequence |
| --- | --- | --- |
| GAPDH | TGTGTCCGTCGTGGATCTGA | TTGCTGTTGAAGTCGCAGGAG |
| CDK2 | AGGTGGAGAAGATTGGAGAGG | CCCGTCAACTTGTTTTTGGCT |
| CDK4 | CCCTCTTCTCACTCTGCGTC | TGCCAGAGATGGAGGAGTCT |
| CDK6 | TGACACTGTGCACACATCAAA | AGACCAGTGAGGAGGGCAT |
| Cyclin D1 | GCGTACCCTGACACCAATCTC | CTCCTCTTCGCACTTCTGCTC |
| Cyclin D2 | GAGTGGGAACTGGTAGTGTTG | CGCACAGAGCGATGAAGGT |
| Cyclin D3 | CGAGCCTCCTACTTCCAGTG | GGACAGGTAGCGATCCAGGT |
| Cyclin G2 | AGGGGTTCAGCTTTTCGGATT | AGTGTTATCATTCTCCGGGGTAG |
| Rb | TGGAATTTGCGACGCTGTCA | CACTTCCTCCCACCCTCCTT |
| P18 | TTATGAAGCACACAGCCTGCAATGT | ACGGACAGCCAACCAACTAACGG |
| P27 | TATGGAAGAAGCGAGTCAGC | GCGAAGAAGAATCTTCTGCAG |
| p16 | ACGGAAAGCCCTGCAATTTAC | TGTGTCTGGAACCTCAATTCTGA |
| p21 | AATCCTGGTGATGTCCGACC | TTGCAGAAGACCAATCTGCG |
| P53 | AAGATCCGCGGGCGTAA | CATCCTTTAACTCTAAGGCCTCATTC |
| EGR1 | TCGGCTCCTTTCCTCACTCA | CTCATAGGGTTGTTCGCTCGG |
| FOS | AGGGGCAAAGTAGAGCAGCTA | CAATCTCAGTCTGCAACGCA |
