# Supplementary figures and images for: Excessive proliferation and impaired function of primitive hematopoietic cells in bone marrow due to senescence post chemotherapy in a T cell acute lymphoblastic leukemia model
Source: J Transl Med. 2015 Jul 17;13:234. doi: 10.1186/s12967-015-0543-8 (PMC4504405; doi:10.1186/s12967-015-0543-8)

## Slide 1
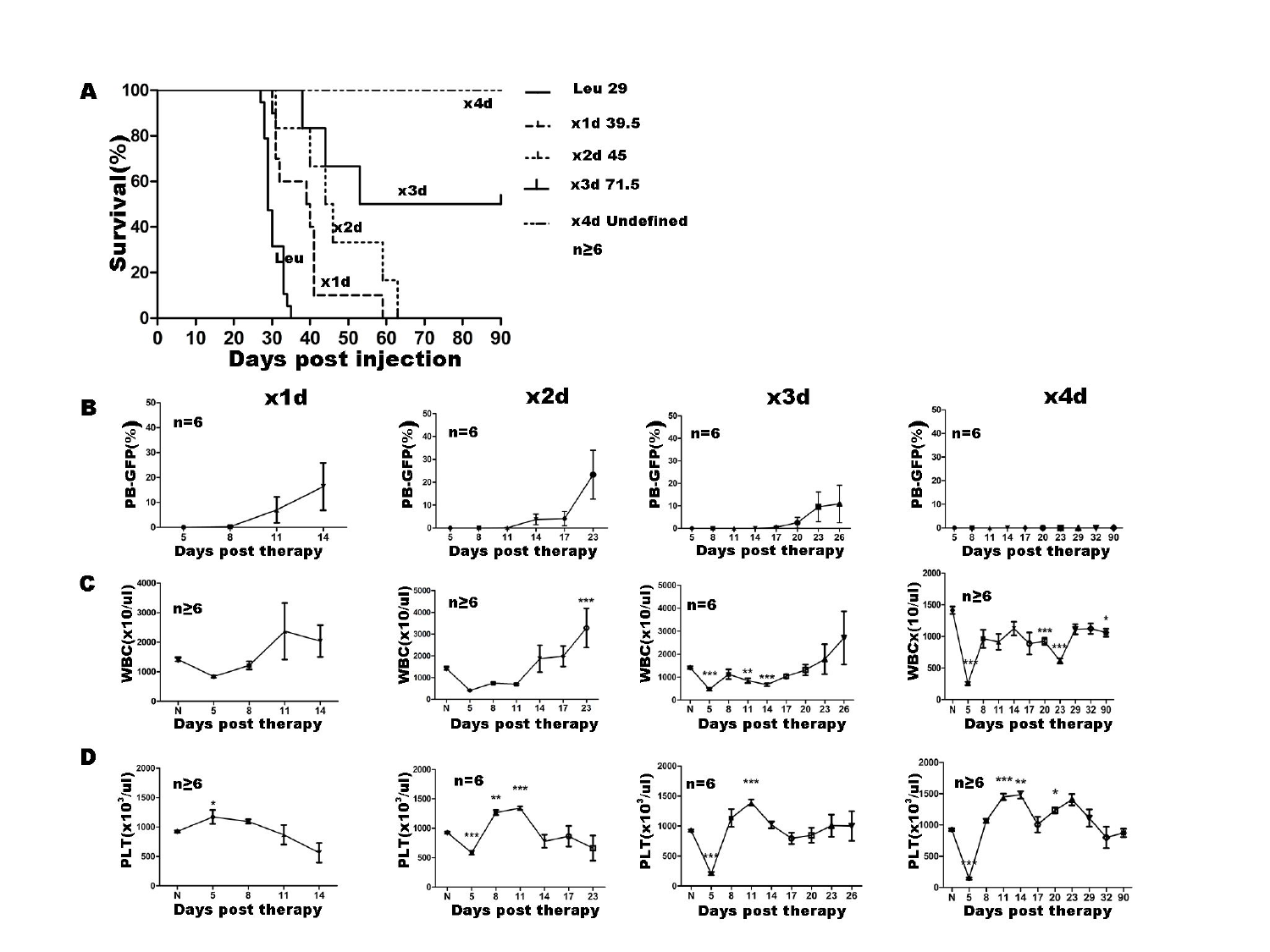

Supplement: Additional file 2: — Course-dependent chemotherapy response of the T-ALL mice. When leukemic cells reached 1-5% in PB, mice received chemotherapy composed of CTX (100 mg/kg) and Ara-C (150 mg/kg) for a consecutive 1, 2, 3 and 4 days, respectively. (A) Median survival days post leukemic cell injection were 29, 39.5, 45 and 71.5 for the leukemia-only, one-day treated, two-day treated and three-day treated group, respectively (n = 6-10). For the four-day treated group, no mice died within the inspecting 90 days (n = 6). (B) Leukemic burden in PB of the four differently treating groups (n = 6). Data showed that longer the therapeutic course, longer the relapse-free period. The day on which leukemic cells showed up again in PB for the one-day, two-day and three-day treated groups were the 8th, 11th and 17th day post therapy, respectively. While for the four-day treated group, no appearance of relapse was detected within the inspecting 90 days. (C) White blood cell count in PB of the four differently treating groups (n = 6). Data showed that longer the therapeutic course, heavier the depression of white blood cells post therapy. (D) Platelet count in PB of the four differently treating groups (n = 6). Data showed that longer the therapeutic course, longer the suppression period of platelet in PB post therapy. All data were presented as mean±SEM. Statistical significance as: * p<0.05; ** p<0.01; *** p<0.001. [file 12967_2015_543_MOESM2_ESM.ppt]

## Slide 1
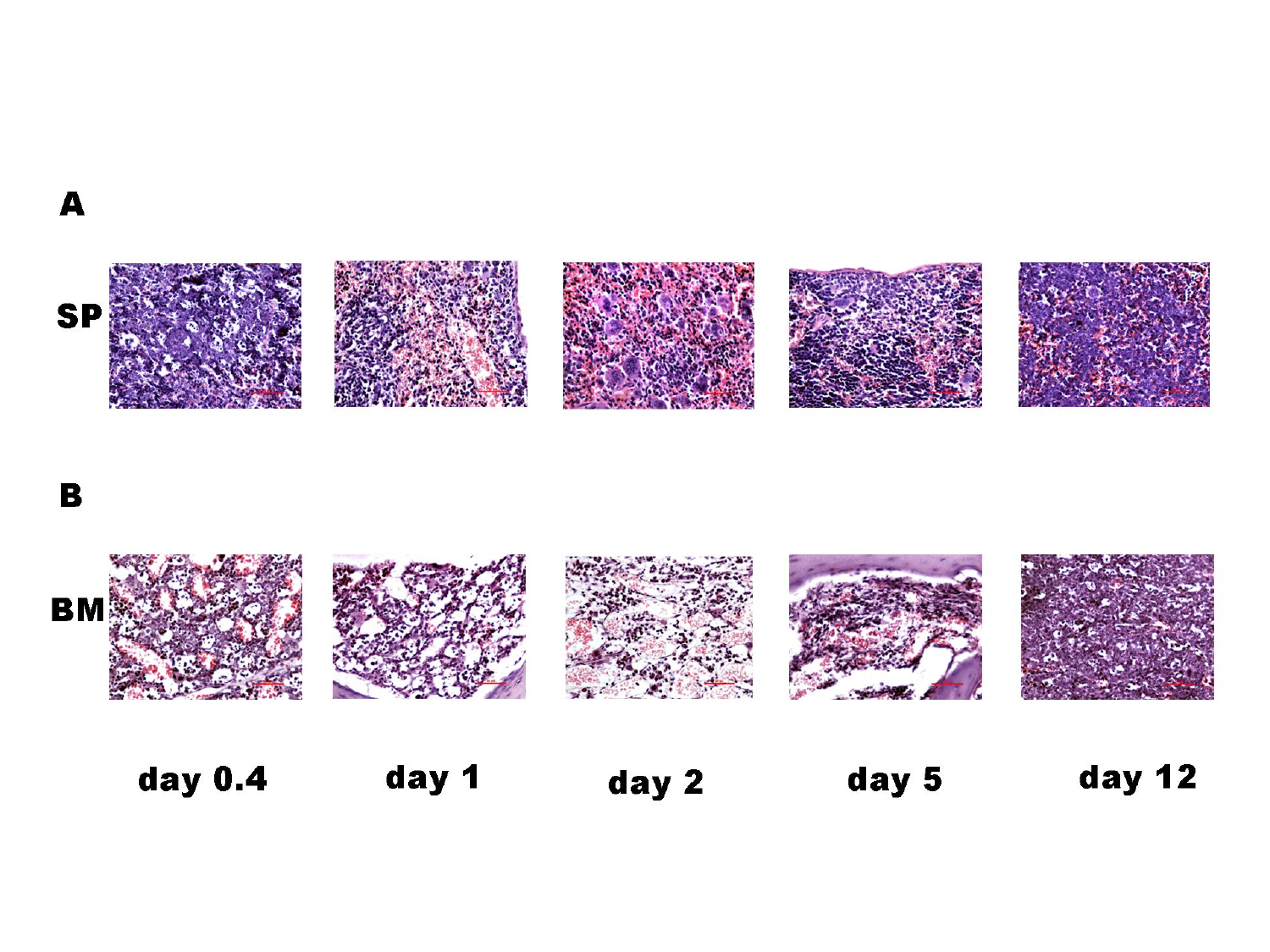

Supplement: Additional file 3: — Histopathology of the one-day treated leukemic mice. Mice of the one-day treated leukemic group were sacrificed at different time points post therapy for histopathological analysis. Tissues were gained right after sacrifice, fixed in 10% formalin overnight, stained by hematoxylin-eosin (H&E) and examined by an inverted microscopy. (A) Status of leukemic infiltration and normal hematopoiesis in spleen post chemotherapy. (B) Status of leukemic infiltration and normal hematopoiesis in bone marrow post therapy. [file 12967_2015_543_MOESM3_ESM.ppt]

## Slide 1
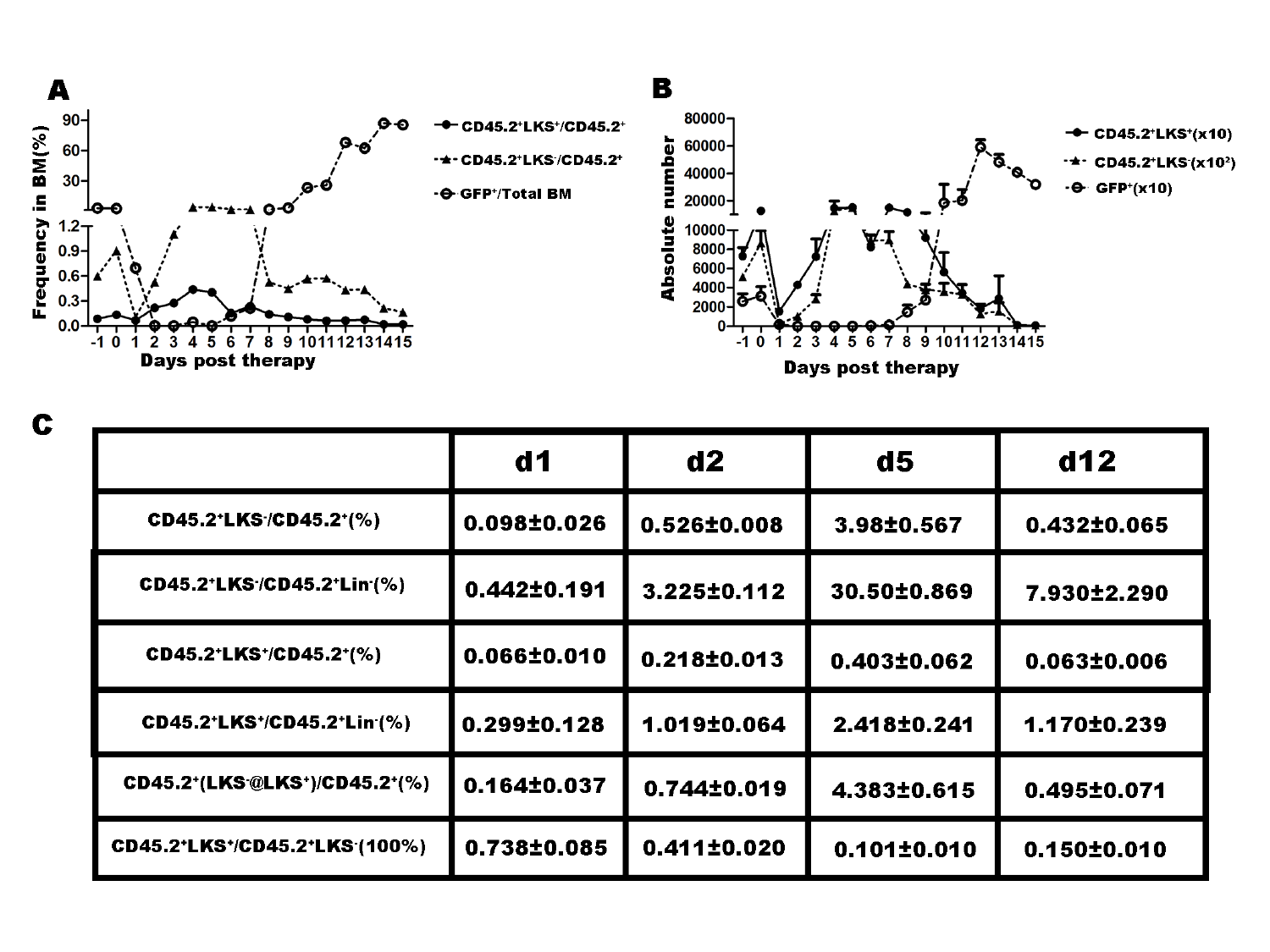

Supplement: Additional file 4: — Kinetics of HSPCs and leukemic cells involved in the one-day treated leukemic group. (A) Frequencies of leukemic cells in whole MNCs and CD45.2+LK+S+ and CD45.2+LK+S− cells in CD45.2+ hematopoietic cell fractions in the BM of the one-day treated leukemic mice (n = 3-4). (B) Total numbers of leukemic cells, CD45.2+LK+S+ and CD45.2+LK+S− cells in BM (double hindlimbs) of the one-day treated leukemic mice (n = 3-4). (C) Statistic analysis of sub-populations of CD45.2+ hematopoietic cell fractions in the one-day treated leukemic mice (n = 3-4). All data were presented as mean ± SEM. [file 12967_2015_543_MOESM4_ESM.ppt]

## Slide 1
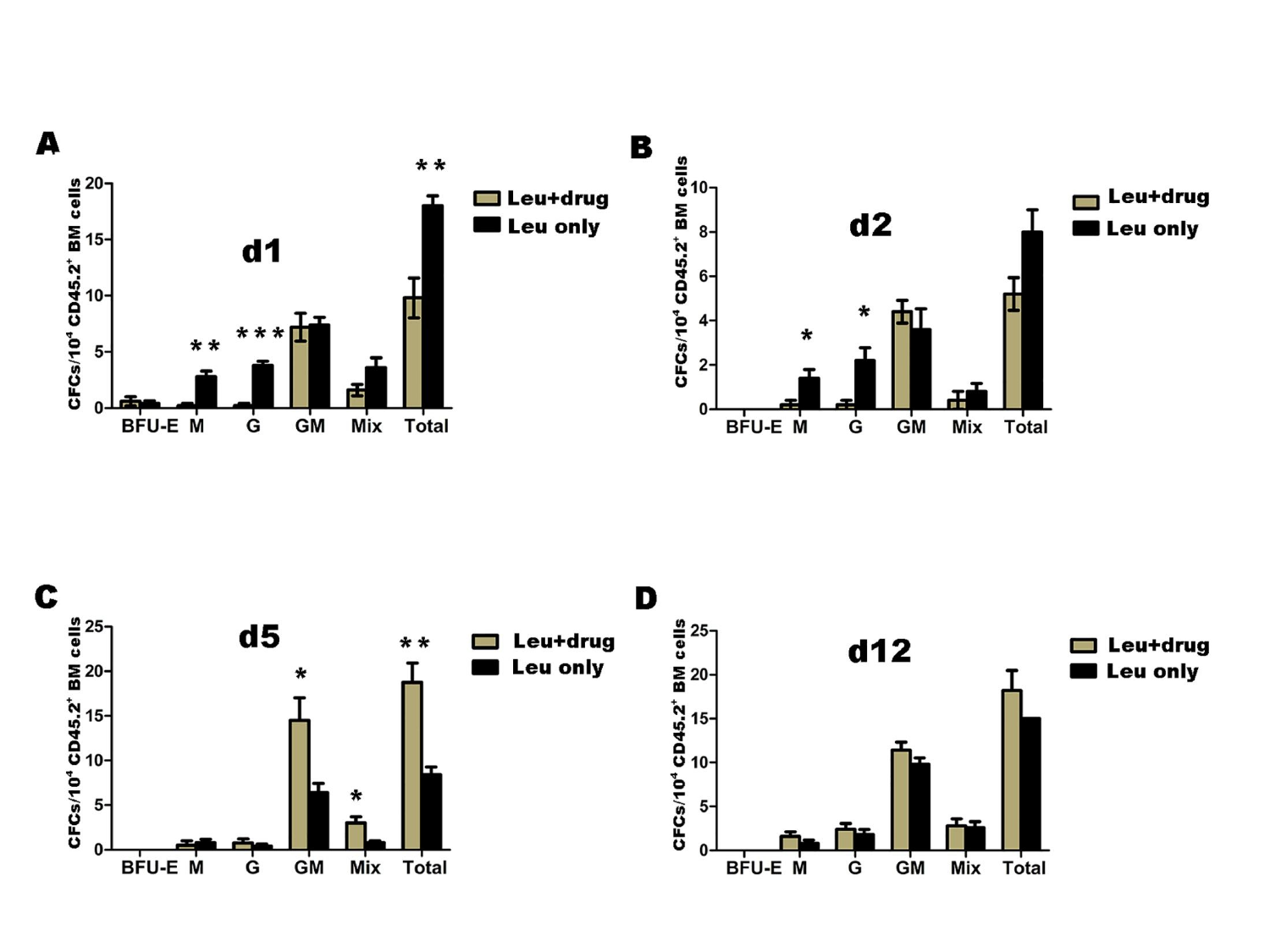

Supplement: Additional file 5: — Results of the in vitro colony-forming cell assays in the one-day treated leukemic mice. CD45.2+ hematopoietic cells in the BM of the one-day treated leukemic mice were sorted for in vitro colony-forming cell assays on different days post therapy. CD45.2+ hematopoietic cells in the BM of leukemia-only mice were used as control. (A–D) Results of the CFC assays (n = 4-5). Data showed similar changing trend of hematopoietic progenitors and the same granulocyte shift in the proliferation phase coincident with flow cytometric data. All data were presented as mean±SEM. Statistical significance as: * p<0.05; ** p<0.01; *** p<0.001. [file 12967_2015_543_MOESM5_ESM.ppt]

## Slide 1
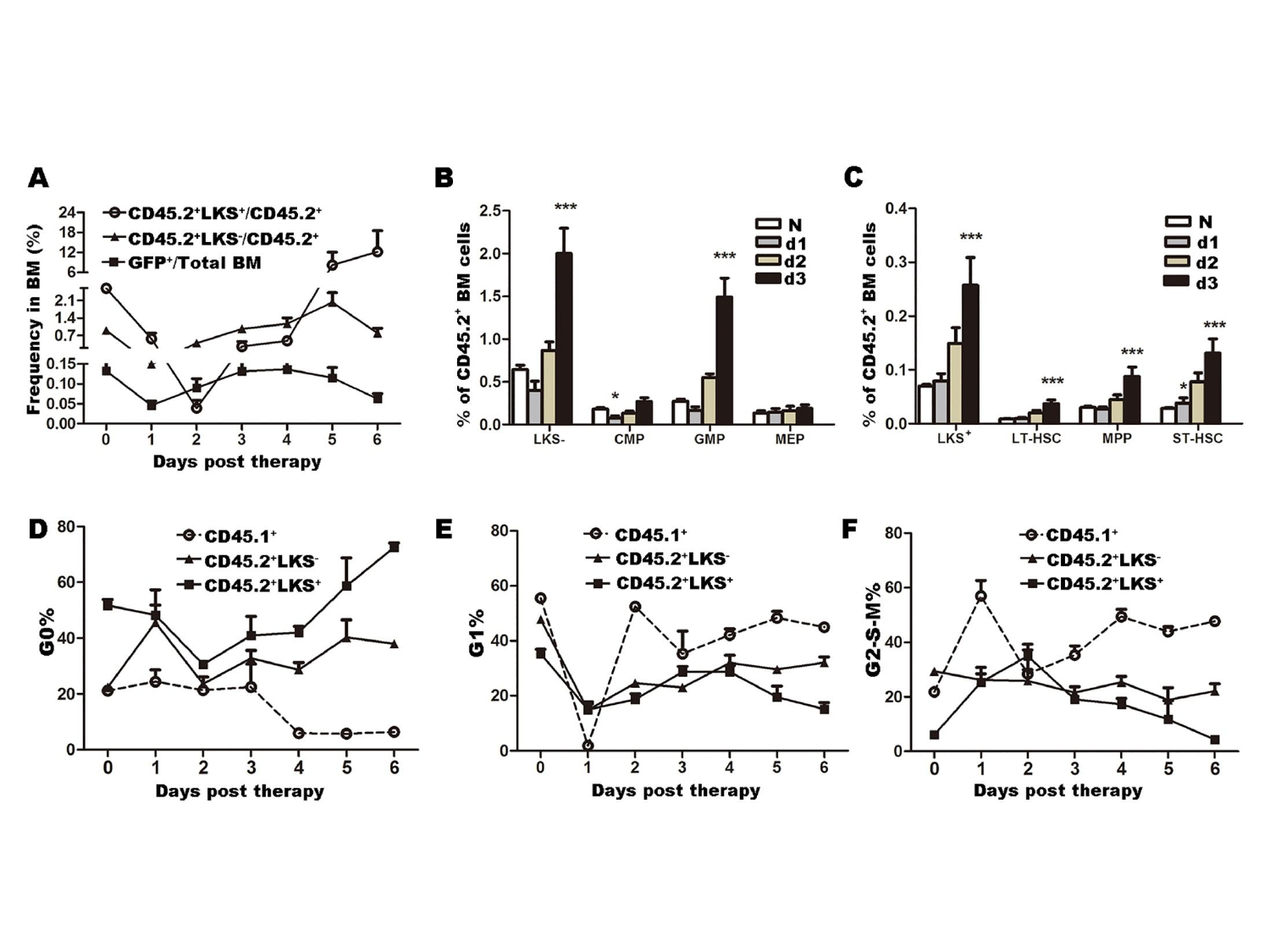

Supplement: Additional file 6: — Kinetics of HSPCs and leukemic cells involved in the lower-dose treated leukemic mice. When leukemic cells reached 1-5% in PB, mice received one-day of lower-dose therapy, composed of 75 mg/kg Ara-C plus 50mg/kg CTX. Then changes of leukemic cells and primitive hematopoietic cell fractions were tested. (A) Frequencies of leukemic cells in whole MNCs and CD45.2+LK+S−, CD45.2+LKS+ cells in CD45.2+ hematopoietic cell fractions in the BM of the lower-dose treated leukemic mice (n = 3-4). (B) Frequencies of LKS− cells, CMP, GMP and MEP in CD45.2+ hematopoietic cell fractions in the BM of normal control and the lower-dose treated leukemic mice on the 1st, 2nd and 3rd day post therapy (n = 5-9). (C) Frequencies of LK+S+ cells, LT-HSC, MPP and ST-HSC in CD45.2+ hematopoietic cell fractions in the BM of normal control and the lower-dose treated leukemic mice on different days post therapy (n = 5-9). (D–F) Cell-cycle status of leukemic cells, CD45.2+LK+S− and CD45.2+LK+S+ cells in the BM of the lower-dose treated leukemic mice on the therapeutic day (d0), and on the six consecutive days since (n = 3-5). All data were presented as mean±SEM. Statistical significance as: * p<0.05; ** p<0.01; *** p<0.001. [file 12967_2015_543_MOESM6_ESM.ppt]

## Slide 1
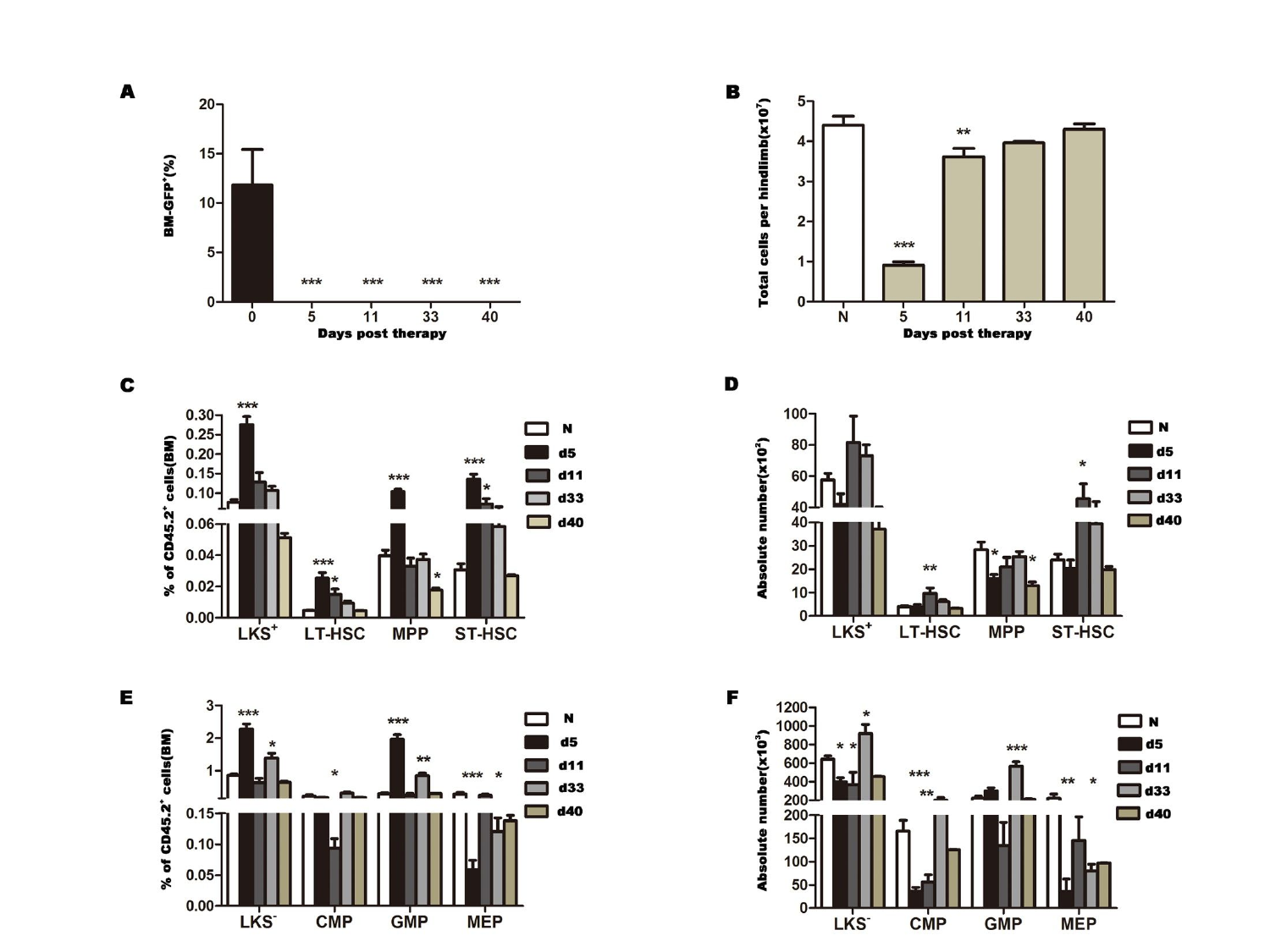

Supplement: Additional file 7: — Kinetics of HSPCs and leukemic cells involved in the four-day treated leukemic group. When leukemic cells reached 1-5% in PB, mice received four consecutive days of treatment, composed of 150 mg/kg Ara-C plus 100 mg/kg CTX daily. Then changes of leukemic cells and primitive hematopoietic cell fractions were tested. (A) Leukemic burden in the BM was continuously undetectable within 40 days post therapy (n = 5). (B) Bone marrow cellularity showed a gradual recovery post therapy (n = 5). (C) Frequencies of LK+S+ cells, LT-HSC, MPP and ST-HSC in CD45.2+ hematopoietic cell fractions in the BM of normal control and the four-day treated leukemic mice post therapy (n = 3-5). (D) Total numbers of hematopoietic LK+S+ cells, LT-HSC, MPP and ST-HSC in the BM (double hindlimbs) of normal control and the four-day treated leukemic mice on different days post therapy (n = 3-5). (E) Frequencies of LKS− cells, GMP, MEP and CMP in CD45.2+ hematopoietic cell fractions in the BM of normal control and the four-day treated leukemic mice on different days post therapy (n = 3-5). (F) Total numbers of hematopoietic LK+S− cells, GMP, MEP and CMP in the BM (double hindlimbs) of normal control and the four-day treated leukemic mice on different days post therapy (n = 3-5). All data were presented as mean±SEM. Statistical significance as: * p<0.05; ** p<0.01; *** p<0.001. [file 12967_2015_543_MOESM7_ESM.ppt]

## Slide 1
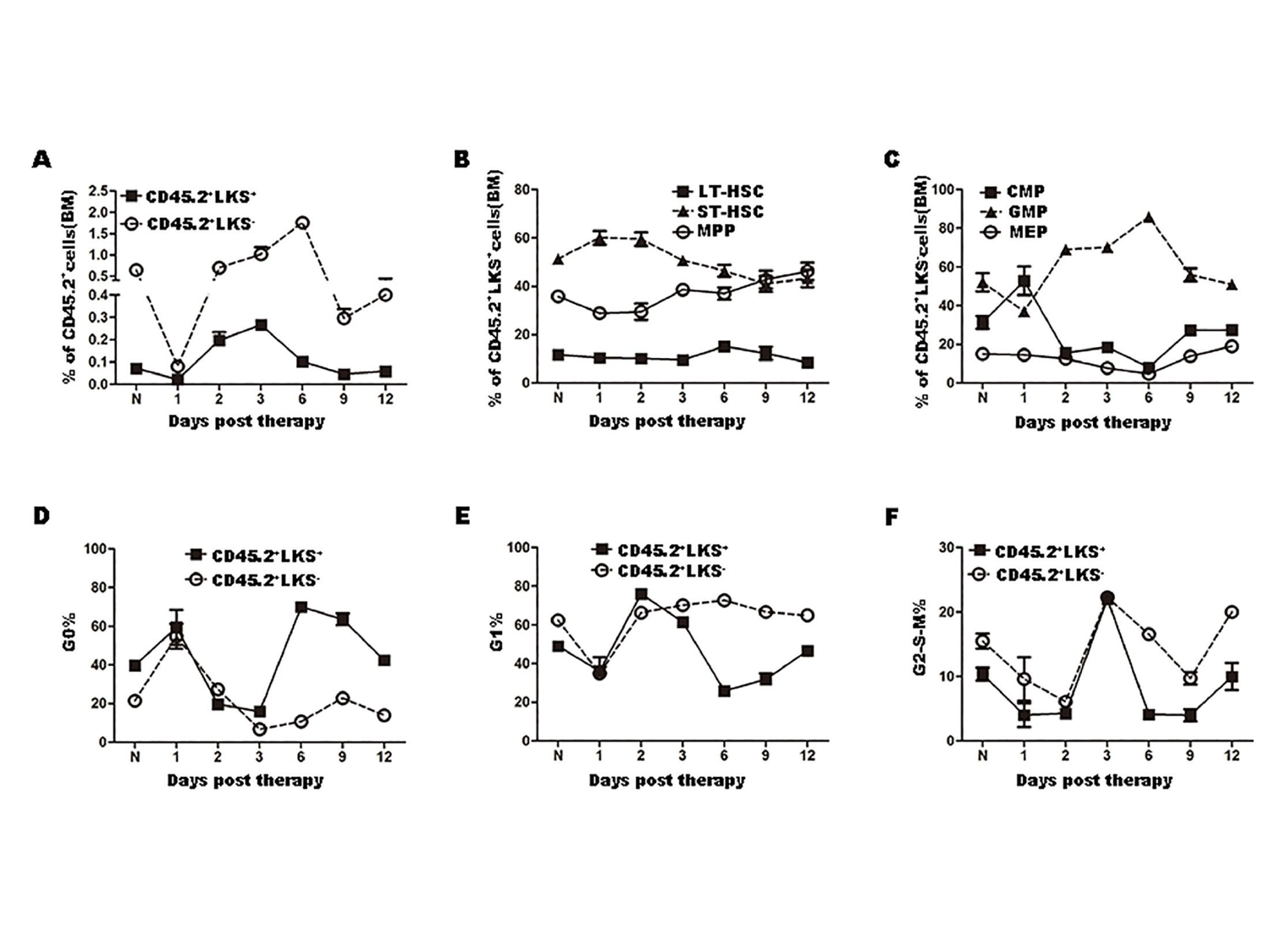

Supplement: Additional file 8: — Kinetics of HSPCs involved in the drug-only group. Normal C57/BL6J mice were given one-day therapy, composed of 150 mg/kg Ara-C plus 100mg/kg CTX. Then changes of primitive hematopoietic cell fractions were tested. (A) Frequencies of CD45.2+LK+S+ and CD45.2+LK+S− cells in the BM CD45.2+ hematopoietic cell fractions of normal control and the drug-only group mice on different days post therapy (n = 3-5). (B) Frequencies of LT-HSC, ST-HSC and MPP in the BM CD45.2+LK+S+ hematopoietic cell fractions of normal control and the drug-only group mice on different days post therapy (n = 3-5). (C) Frequencies of CMP, GMP and MEP in the BM CD45.2+LK+S− hematopoietic cell fractions of normal control and the drug-only group mice on different days post therapy (n = 3-5). (D) Frequencies of BM CD45.2+LK+S+ and CD45.2+LK+S− cells in G0 phase of normal control and the drug-only group mice on different days post therapy (n = 3-5). (E) Frequencies of BM CD45.2+LK+S+ and CD45.2+LK+S− cells in G1 phase of normal control and the drug-only group mice on different days post therapy (n = 3-5). (F) Frequencies of BM CD45.2+LK+S+ and CD45.2+LK+S− cells in G2-S-M phase of normal control and the drug-only group mice on different days post therapy (n = 3-5). All data were presented as mean ± SEM. Statistical significance as: * p<0.05; ** p<0.01; *** p<0.001. [file 12967_2015_543_MOESM8_ESM.ppt]
